# Accurate clinical toxicity prediction using multi-task deep neural nets and contrastive molecular explanations

**DOI:** 10.1038/s41598-023-31169-8

**Published:** 2023-03-25

**Authors:** Bhanushee Sharma, Vijil Chenthamarakshan, Amit Dhurandhar, Shiranee Pereira, James A. Hendler, Jonathan S. Dordick, Payel Das

**Affiliations:** 1 Chemical and Biological Engineering, RPI, Troy, NY USA; 2 IBM Research, Yorktown Heights, NY USA; 3 ICARE, International Center for Alternatives in Research and Education, Chennai, India; 4 Computer Science, RPI, Troy, NY USA

**Keywords:** Toxicology, Machine learning

## Abstract

Explainable machine learning for molecular toxicity prediction is a promising approach for efficient drug development and chemical safety. A predictive ML model of toxicity can reduce experimental cost and time while mitigating ethical concerns by significantly reducing animal and clinical testing. Herein, we use a deep learning framework for simultaneously modeling in vitro, in vivo, and clinical toxicity data. Two different molecular input representations are used; Morgan fingerprints and pre-trained SMILES embeddings. A multi-task deep learning model accurately predicts toxicity for all endpoints, including clinical, as indicated by the area under the Receiver Operator Characteristic curve and balanced accuracy. In particular, pre-trained molecular SMILES embeddings as input to the multi-task model improved clinical toxicity predictions compared to existing models in MoleculeNet benchmark. Additionally, our multitask approach is comprehensive in the sense that it is comparable to state-of-the-art approaches for specific endpoints in in vitro, in vivo and clinical platforms. Through both the multi-task model and transfer learning, we were able to indicate the minimal need of in vivo data for clinical toxicity predictions. To provide confidence and explain the model’s predictions, we adapt a post-hoc contrastive explanation method that returns pertinent positive and negative features, which correspond well to known mutagenic and reactive toxicophores, such as unsubstituted bonded heteroatoms, aromatic amines, and Michael receptors. Furthermore, toxicophore recovery by pertinent feature analysis captures more of the in vitro (53%) and in vivo (56%), rather than of the clinical (8%), endpoints, and indeed uncovers a preference in known toxicophore data towards in vitro and in vivo experimental data. To our knowledge, this is the first contrastive explanation, using both present and absent substructures, for predictions of clinical and in vivo molecular toxicity.

Toxicity remains a major driver of drug candidate failure in drug development, resulting in the high cost of drugs that make it into the market^1,2^. This phenomenon has persisted despite the surge in new chemical entities (NCEs) resulting from both advances in omics technology and the ability of Machine Learning (ML) models to generate novel molecules^3–5^. Consequently, there is an increasing need to accurately and efficiently predict the safety of new drug candidates in humans. To this end, there has been an escalation of ML models predicting toxicity, but not without its challenges. Of the many challenges, one is to correctly model a multi-faceted problem across different in vitro, in vivo and clinical platforms of varying granularities. Another non-trivial challenge is to comprehensively explain predictions across all these platforms which is non-trivial. To illustrate on the first challenge, a variety of ML models have been applied to chemical, biological, and mechanistic data that predict the toxicity of chemicals with varying granularity and relevancy to toxicity in humans, i.e., clinical toxicity (Section 1 in Supplementary). These models have differed in inputs^6–10^, architectures^8,11–15^, and prediction platforms (i.e., endpoints or the specific experimental target including in vivo, in vitro, or clinical). A majority of ML models have focused on predicting specific in vitro endpoints^16–19^ from only chemical structures , differing mainly in the molecular representations used^8–10,20^. In particular, nuclear receptor endpoints were a major focus of the Tox21 Challenge, a data challenge as a subset of the broader “Toxicology in the 21st Century” initiative. The Tox21 challenge provides the results of 12 in vitro assays that test seven different nuclear receptor signaling effects and five stress response effects of 10,000 molecules in cells^16–19^.

Toxicities predicted in vitro or in vivo are not necessarily in concordance with each other^21,22^ nor to humans^2,23–26^, thus reducing their ability to predict clinical toxicity^2^. The granularity of toxicity tests varies across the in vitro, in vivo, and clinical platforms. In vitro testing is the most granular and captures the ability of a chemical to disrupt biological pathways at the cellular level. In contrast, clinical testing is coarse-grained and captures the interactions of chemicals at multiple levels in the human, including organs and tissues. Thus, ML models trained on in vitro and in vivo data might not reliably capture clinical toxicity.

Despite toxicity being a multi-task problem, majority of ML models have predicted toxicity in each platform separately with single-task models (Section 1 in Supplementary). A single molecule can demonstrate simultaneously a multitude of responses in different assays and different living organisms. Various solutions for modeling multiple toxic endpoints have been reported, by creating separate binary classification models for each endpoint^7,27^, or by using multiple classification models that define classes differently^28–36^. Yet, thus far, the multiplicity problem has been modeled by predicting multiple endpoints within the same testing platform: in vitro, in vivo, or in humans, separately.

The second challenge of ML models in predictive toxicology is to explain the predictions made, particularly within deep learning-based models. ML toxicity models have increasingly shifted towards deep learning^8,15^ pushed by its superior predictive performance^8^ and ability to self-select significant features^37^. Deep learning (DL) models are “black-box” models with limited explainability, i.e., do not provide reasoning for predicting that a molecule is toxic or nontoxic. This explanation is essential for designing new molecules and for providing greater confidence to the experimentalist end-users. As a result, the Organisation for Economic Co-operation and Development (OECD) strongly recommends that predictions of computational toxicology models be explainable^38^. Efforts in explaining toxicity predictions have focused on pinpointing the presence of certain features, such as toxicophores, derived from a range of methods, including simpler quantitative structure-activity relationship (QSAR) models^39,40^ and explaining training in Deep Neural Networks (DNNs)^8^ (Section 1.1 in Supplementary). These methods generally do not examine the effect of the absence of these features. Defining minimal and necessary features, as well as present and absent features, might provide a comprehensive explanation that is more intuitive to end-users.

Herein, we have developed a deep learning framework with the aim of improving accuracy and explainability of clinical toxicity predictions by taking advantage of in vitro, in vivo, and clinical toxicity data, and more advanced molecular representations. We simultaneously predicted in vitro, in vivo, and clinical toxicity through deep multi-task models, while comparing to their single-task and transfer learning counterparts. Two different molecular representations were tested; Morgan fingerprints and in-house created pre-trained SMILES embeddings encoding for the relationships among the chemicals. We used this framework for establishing concordance across in vitro, in vivo, and clinical datasets. Notably, in response to the adoption of the 3 Rs (Replacement, Reduction, and Refinement of animal testing) in global legislation^41,42^, we assessed the need for animal data in making clinical toxicity predictions. To provide more comprehensive molecular explanations of toxicity predictions, we adopted the Contrastive Explanations Method (CEM) explainability model^43^ which explains “black-box” DNN predictions by revealing pertinent positive (PP) and pertinent negative (PN) features as chemical structures correlating to a given prediction. Specifically, we explained single-task toxicity predictions in vitro, in vivo, and clinically. The PPs represent the minimum required substructures for classification of a molecule (toxicophores for a toxic prediction), and the PNs represent the minimum changes to a molecule that would flip its predicted class label, from toxic to nontoxic or vice versa. Such an explanation should expand the scope of explainability in predictive toxicology while providing information on both toxicophores and nontoxic substructures.

## Model framework

**Figure 1:**
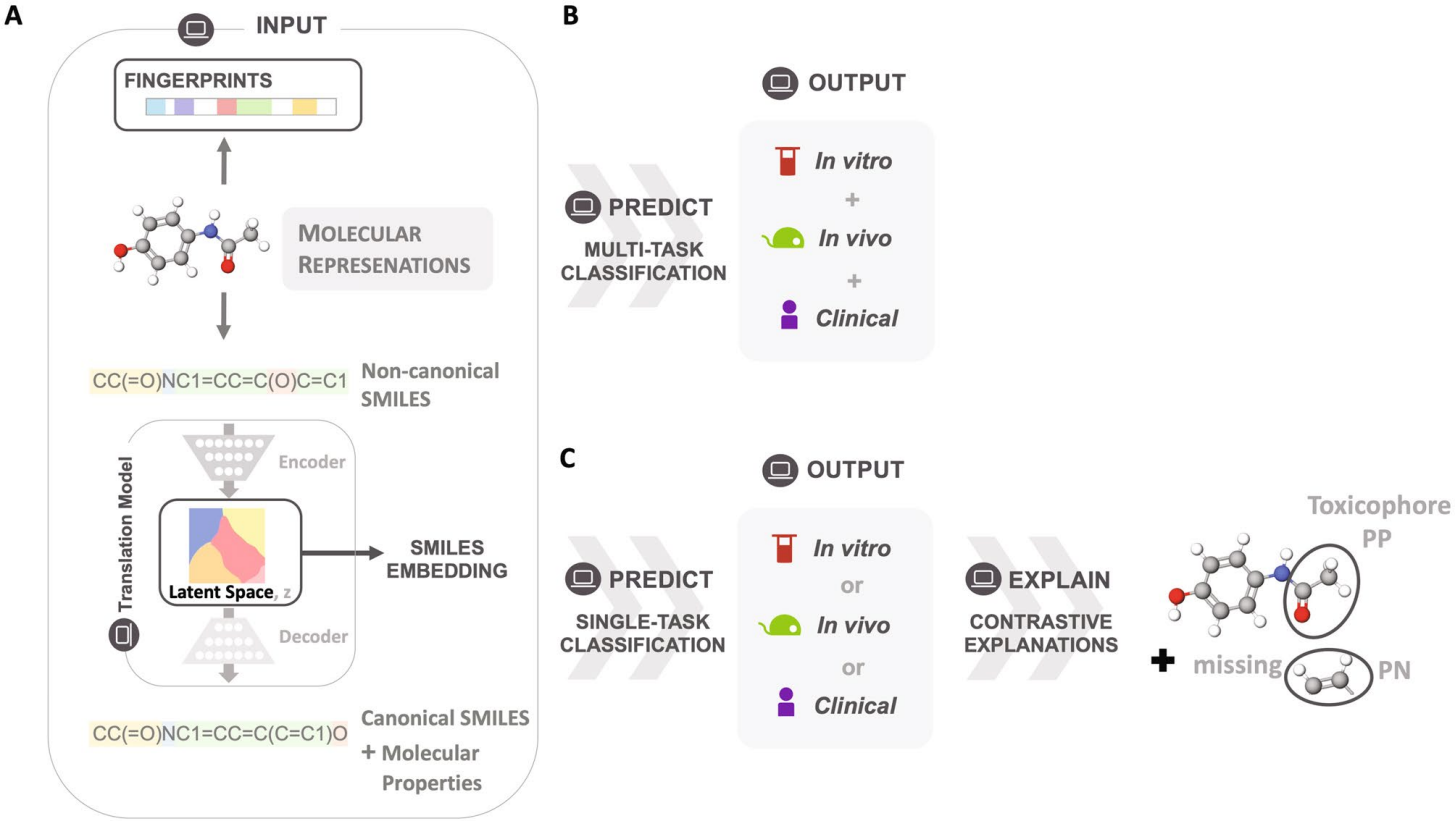
Framework adopted in this study for explainable, single-task, and multi-task prediction of in vitro, in vivo, and clinical toxicity. (**A**) Given an input of different molecular representations, fingerprints and latent space SMILES embeddings, (**B**) a multi-task classification model predicts whether a molecule is toxic or not for in vitro, in vivo, and clinical endpoints. Furthermore, the Contrastive Explanation method explains (**C**) predictions from single-task models trained on fingerprints for the same endpoints. The method pinpoints minimal and necessary chemical substructures that are either present (pertinent positive, PP) or absent (pertinent negative, PN) for a specific prediction.

Two different molecular representations were used as inputs; commonly used Morgan Fingerprints (FP) and more complex SMILES embeddings (SE) (Fig. 1A). FP vectorize the presence of a substructure within varying radii around an atom. FP are easy to compute and have high performance among other fingerprints^44^, and are thus widely used. However, FP are simplistic representations of chemical structures, not coding for relationships between substructures, unlike molecular graphs, nor for relationships between the chemicals. To improve on this, the SE were created using a neural network-based model that translates from non-canonical SMILES to canonical SMILES, encoding for the relationship between chemicals within the datasets.

With these molecular representations, toxicities in vivo, in vitro, and clinical, were predicted through multi-task (MTDNN) (Fig. 1B) and single-task Deep Neural Networks (STDNN) (Fig. 1B). The MTDNN predicts each platform (in vivo, in vitro, clinical) as a different task within one model, with each task consisting of a single or multiple classes (or endpoints). In contrast, the STDNN predicts each platform with a separate model.

As a proof-of-concept, endpoints from previous benchmarking efforts^45^ and data challenges^16^ were chosen, and are not exhaustive. For the clinical platform, the endpoint was whether or not a molecule failed clinical phase trials due to toxicity, as obtained from the ClinTox dataset^45^. For the in vitro platform, 12 different endpoints from the Tox21 Challenge^16^ were used, whether or not a molecule is active in disrupting seven neural receptor assays and five stress response assays. Finally, for the in vivo platform, one endpoint for acute oral toxicity in mice was used from the commercially available RTECS (Registry of Toxic Effects of Chemical Substances) dataset. The acute oral toxicity endpoint was defined by an LD_50_ (lethal dose for 50% of the population) cutoff of 5000 mg/kg specified by GHS and EPA (< 5000 mg/kg as toxic, > 5000 mg/kg as nontoxic).

We further tested the need of in vivo data to predict clinical toxicity with the multi-task DNN and its transfer learning counterpart. Different combinations of in vivo, in vitro, and clinical tasks in the MTDNN were investigated to determine the most relevant tasks for predicting clinical toxicity. One way to achieve this is to leverage transfer learning, which allows a base model trained on one task to be re-purposed for another related task. In our use case, we compared the ability of a base model trained on in vivo or in vitro data to be transferred to predicting clinical toxicity.

Further, we explained DNN predictions by adapting the Contrastive Explanations Method (CEM)^43^ to molecular structure input. Specifically, the CEM was adapted to explain toxicity predictions made by the STDNN trained on Morgan fingerprints for in vivo, in vitro, and clinical platforms (Fig. 1C). For an overview of related work on explanations for molecular toxicity prediction see Section 1.1 in Supplementary. We had previously performed a proof-of-concept^46^ for this approach by explaining predictions on one specific in vitro endpoint (“SR-MMP”, the ability of molecules to disrupt the mitochondrial membrane potential (MMP) in cells^16^). In this work, we further expand to other in vitro endpoints, and to in vivo and clinical platforms. The CEM explains by identifying pertinent positive (PP) and counterfactual pertinent negative (PN) substructures within the input molecules. The PPs are the minimal and necessary substructures that correlate to a prediction, while the PNs are substructures that would switch the given prediction.

## Results

### Deep single-task and multi-task predictive models: Morgan fingerprints and SMILES embeddings

**Figure 2:**
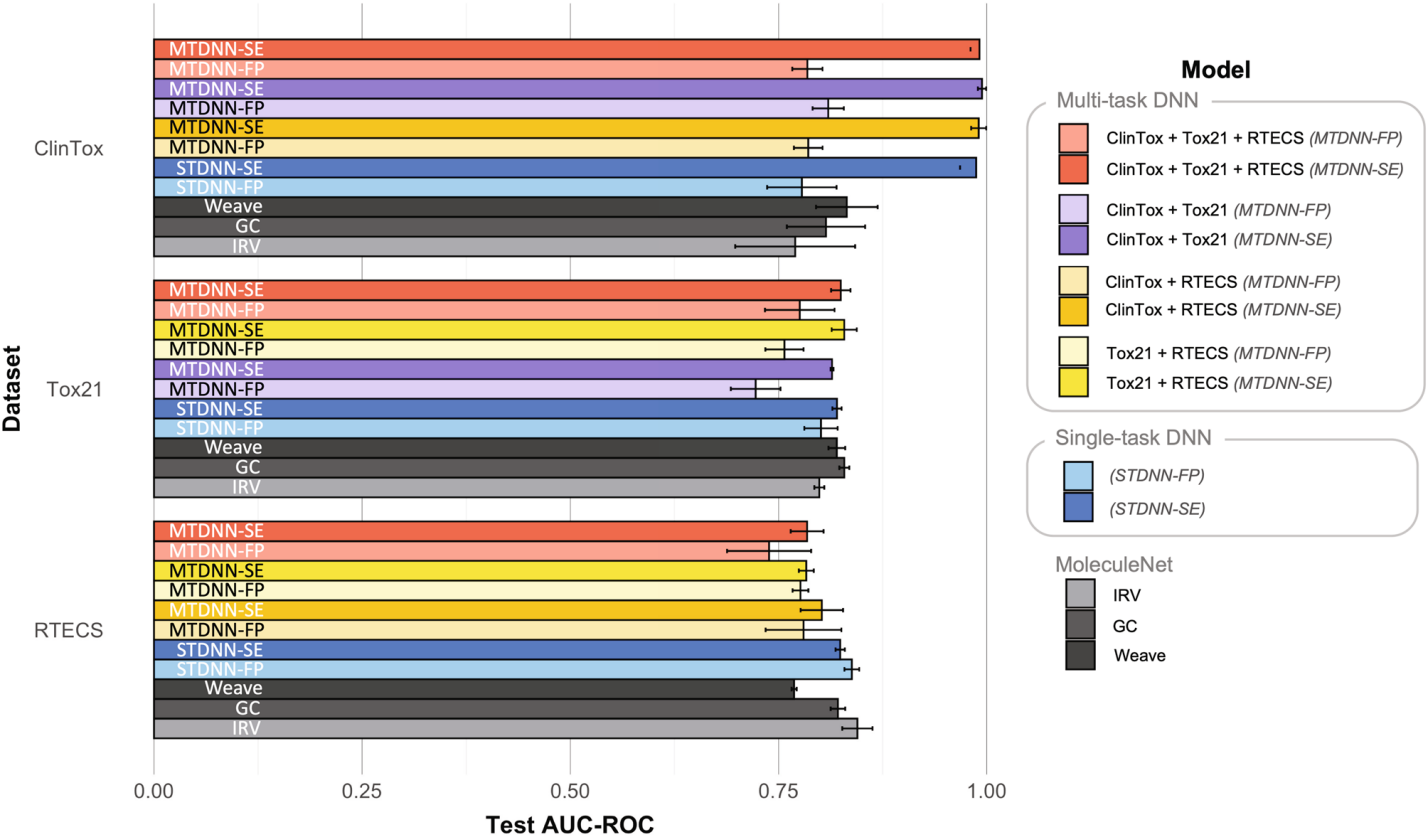
Test AUC-ROC values for ClinTox, Tox21, and RTECS predictions, comparing multi-task models to single-task and baseline MoleculeNet models, with SMILES embeddings and Morgan fingerprints as inputs. The best performing model on ClinTox, Tox21, and RTECS from MoleculeNet is displayed. All other MoleculeNet models are in Supplementary Fig. S2.

**Figure 3:**
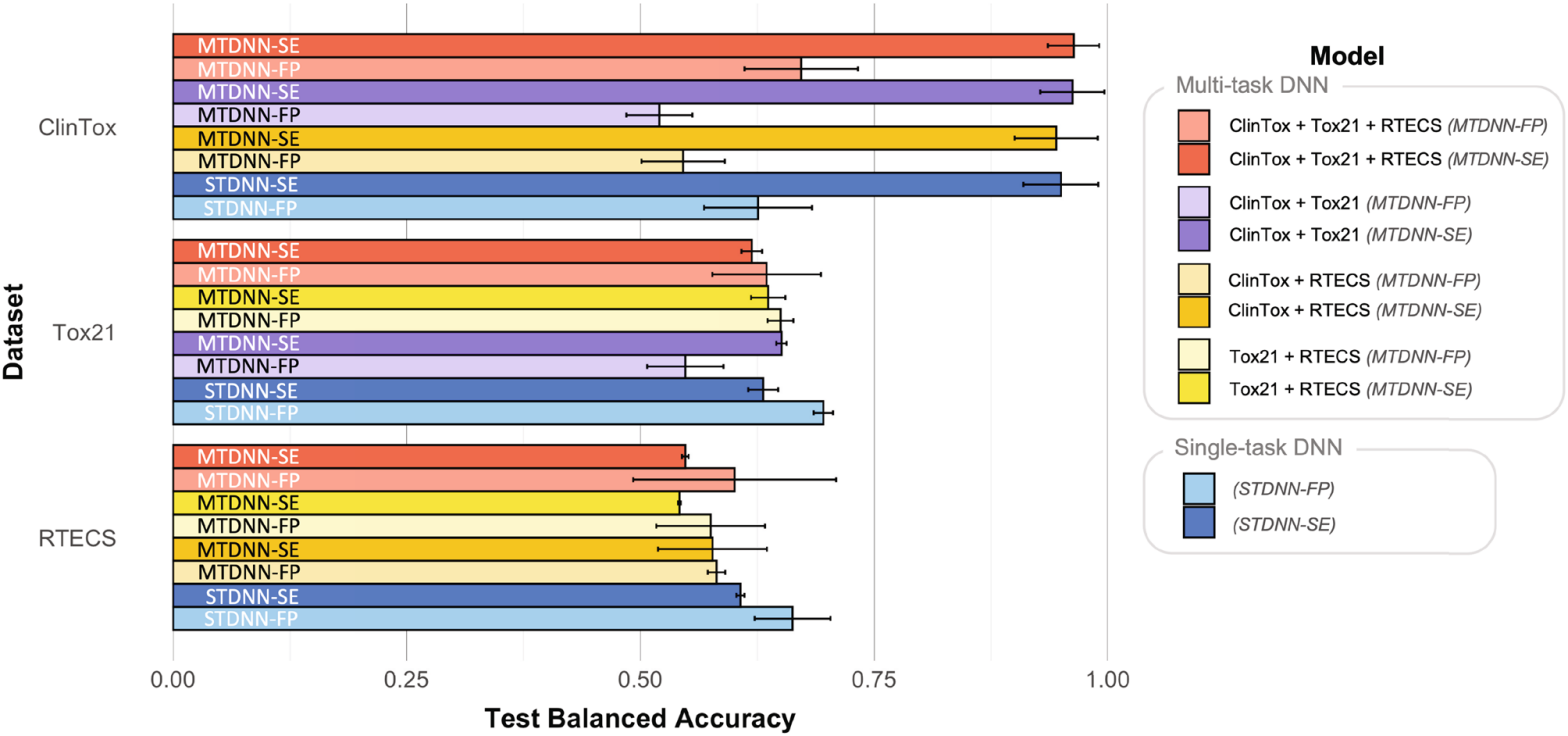
Average balanced accuracy on the test set for ClinTox, Tox21 and, RTECS predictions, comparing multi-task models to single-task models, with SMILES embeddings and Morgan fingerprints as inputs.

We evaluated the performance of our framework by metrics of Area under Receiver Operating Characteristic curve (AUC-ROC) and balanced accuracy. We compared the performance of STDNN (blue in Figs. 2, 3) to MTDNN (multiple colors in Figs. 2, 3) with either SMILES embeddings (SE, darker color in Figs. 2, 3) or Morgan fingerprints (FP, lighter colors in Figs. 2, 3) as input. Performance of different platform combinations in the MTDNN was contrasted by combining all three in vivo, in vitro and clinical platforms (red in Figs. 2, 3), clinical and in vitro platforms (purple in Figs. 2, 3), clinical and in vivo platforms (orange in Figs. 2, 3), and in vitro and in vivo platforms (yellow in Figs. 2, 3).

Area under the ROC curves (AUC-ROC) was determined against the best performing models in MoleculeNet^34^ (grey in Fig. 2), a widely used benchmark for molecular predictions. The best model employed by MoleculeNet on ClinTox (clinical) was a graph neural net baseline operating on molecular graphs^47^ (Weave). On Tox21 (in vitro), the best performing model in Moleculenet was a graph convolutional neural net (GC)^34^. MoleculeNet did not benchmark RTECS (in vivo), but we used their provided benchmark methods to train and test the models used on ClinTox and Tox21^34^, on RTECS. The resulting best model from MoleculeNet on RTECS was the influence relevance voting system (IRV), which is an enhanced k-nearest neighbor model augmented by weights provided from one-layer DNNs^34^.

#### Area under receiver operating characteristic curve

AUC-ROC performance markedly improved on predicting clinical toxicity (ClinTox) using the combination of SE and MTDNN. Compared to Weave, the best performing baseline model on ClinTox (dark grey in Fig. 2), the single-task DNN with SE (STDNN-SE, dark blue in Fig. 2) improved AUC-ROC values from $0.832 \pm 0.037$ to $0.987 \pm 0.019.$ The multi-task DNN trained with SE (MTDNN-SE) further improved AUC-ROC performance on ClinTox with similar values when trained on all three platforms ($0.991 \pm 0.011,$ dark red in Fig. 2) or trained on ClinTox and Tox21 ($0.994 \pm 0.005,$ dark purple in Fig. 2). For predicting in vitro toxicity (Tox21), the MTDNN and STDNN models performed similarly to MoleculeNet’s GC model ($0.829 \pm 0.006,$ grey in Fig. 2) when using SE as input. STDNN-SE gave AUC-ROC of $0.820 \pm 0.006$ (dark blue in Fig. 2). The MTDNN-SE performed comparably trained on all three platforms ($0.825 \pm 0.012,$ dark red in Fig. 2) and on Tox21 and RTECS ($0.829 \pm 0.015,$ dark yellow in Fig. 2). Interestingly, for the in vivo endpoint (RTECS), neither the multi-task model nor the use of the SE showed improved performance when compared to the fingerprint-based single-task models (light blue in Fig. 2) or MoleculeNet’s IRV model (light grey in Fig. 2). Thus, the MTDNN-SE appeared to improve clinical toxicity predictions and provided comparable performance on Tox21, but not on RTECS.

#### Balanced accuracy

Skewed datasets are a prevalent problem in predictive toxicology^7,48,49^. Regardless of the platform, the distribution of toxic and nontoxic examples is often imbalanced (Supplementary Fig. S3). Within the datasets studied here, the imbalance is biased towards the “nontoxic” class in ClinTox and Tox21, and the “toxic” class in RTECS (Fig. S4 in Supplementary for single-task models, and Supplementary Fig. S5 for multi-task models). This biases the AUC-ROC values towards a small fraction of true toxic or true nontoxic predictions. The balanced accuracy (BA) metric takes into account this imbalance and has been used as a more representative metric for predictive toxicology models^7,48,49^. Balanced accuracy averages the sensitivity and the specificity. The former is the fraction of correctly classified positive classes out of all possible positives in the dataset, i.e., fraction of true positives out of correctly classified positives and falsely classified negatives. Conversely, the specificity is this measure for the true negatives of the model, i.e., the fraction of true negatives correctly classified out of the total number of negatives in the dataset (both the correctly classified negatives and the falsely classified positives). However, current baseline models in MoleculeNet do not provide balanced accuracy performance on the toxicity benchmarks.

We report balanced accuracy for all three platforms (Fig. 3). High balanced accuracy, 0.95–0.96, was achieved by both MTDNN-SE and STDNN-SE models for clinical toxicity predictions (ClinTox, dark colors in Fig. 3). The best balanced accuracy on the clinical task was given by the MTDNN-SE trained on all three platforms ($0.963 \pm 0.028,$ dark red in Fig. 3). The DNN models also resulted in a balanced accuracy of $\sim$ 0.64 on Tox21 and RTECS predictions, which is still notably better than random classification (balanced accuracy of 0.5) (Fig. 3). Overall, use of both the multi-task setting and SMILES embeddings helped improve the balanced accuracy on the clinical platform much more so than on in vitro or in vivo.

### Testing the relative importance of in vivo data to predict clinical toxicity

The relative importance for in vivo data for predictions of clinical toxicity was assessed by training different combinations of platforms in the multi-task model, with or without in vivo data, and its transfer learning counterpart. For clinical toxicity predictions (ClinTox), MTDNN models without in vivo data (purple in Fig. 2) performed better in terms of AUC-ROC values when compared to models with in vivo data (red or orange in Fig. 2). To further examine this correlation, the distribution of true/false positives and true/false negatives of clinical toxicity predictions was determined using true labels based on in vitro or in vivo datasets (Supplementary Fig. S6). Conclusions on this correlation are difficult due to the small number of chemicals common across the platforms. From the small overlap of chemicals, a larger number of true positives than false positives was determined for the predictions on ClinTox using true labels from Tox21 than using RTECS (Supplementary Fig. S6). The SR-p53 in vitro assay (stress response on p53) in Tox21 was the sole exception.

**Figure 4:**
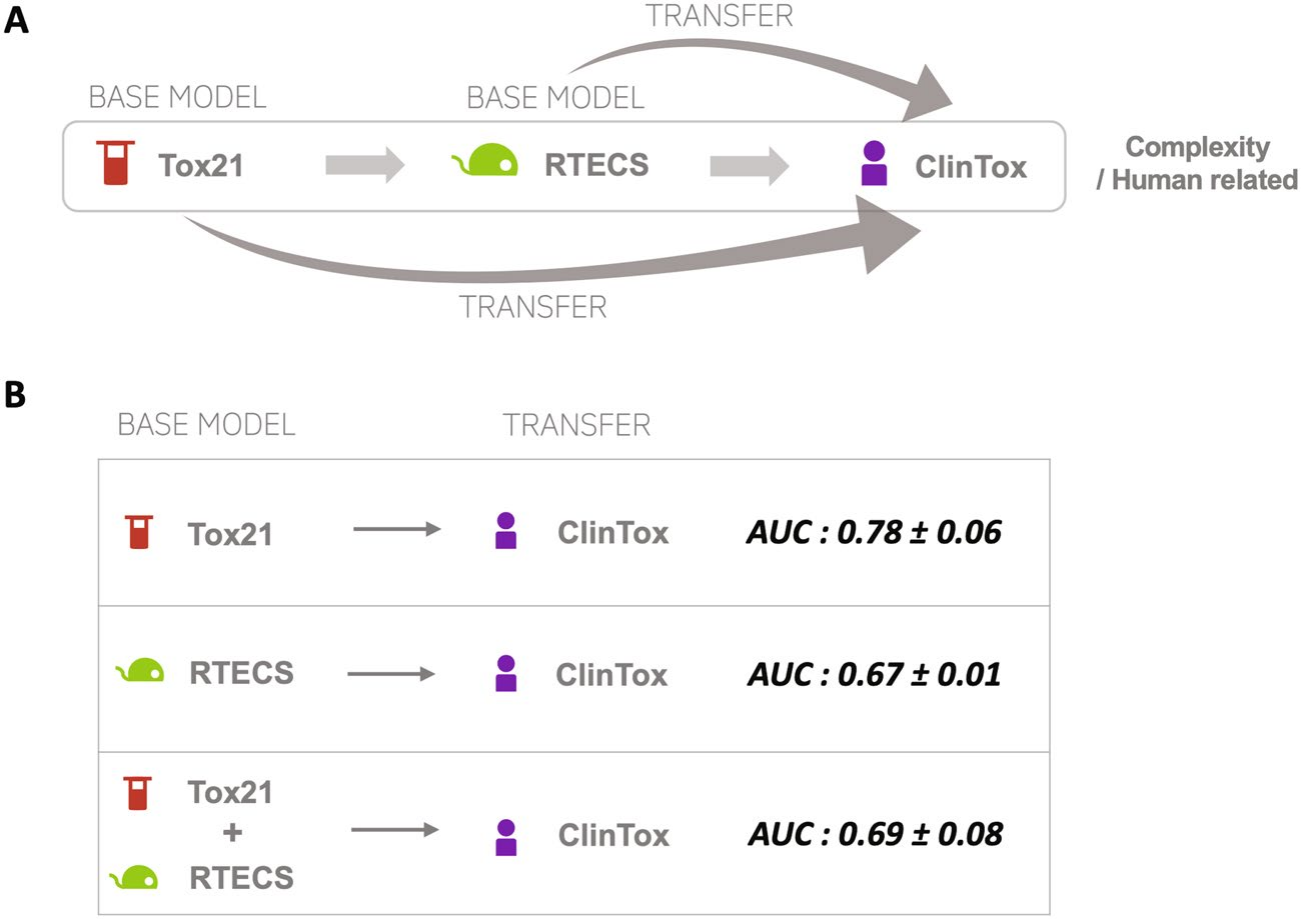
Comparing transfer learning to predicting clinical toxicity from base in vitro and in vivo models. (**A**) Schematic of transfer learning model, with a base model pre-trained on either on in vitro or in vivo data transferred to predicting clinical toxicity. (**B**) AUC results on the ClinTox (clinical) tasks with one epoch training during transfer learning to ClinTox tasks.

Transfer learning applies knowledge gained from pre-training on a base model to predicting in a related domain^50^. We contrasted the application of base models trained on in vivo or in vitro data to predicting clinical toxicity (Fig. 4A). A base model trained with in vivo (RTECS) data decreased in AUC-ROC performance on ClinTox (clinical) when compared to a base model containing only in vitro (Tox21) data (AUC of 0.78 ± 0.06 versus 0.67 ± 0.01 or 0.69 ± 0.08) (Fig. 4B). Thus, pre-training with an in vitro base model transfers to predicting clinical toxicity better than with an in vivo base model.

**Figure 5:**
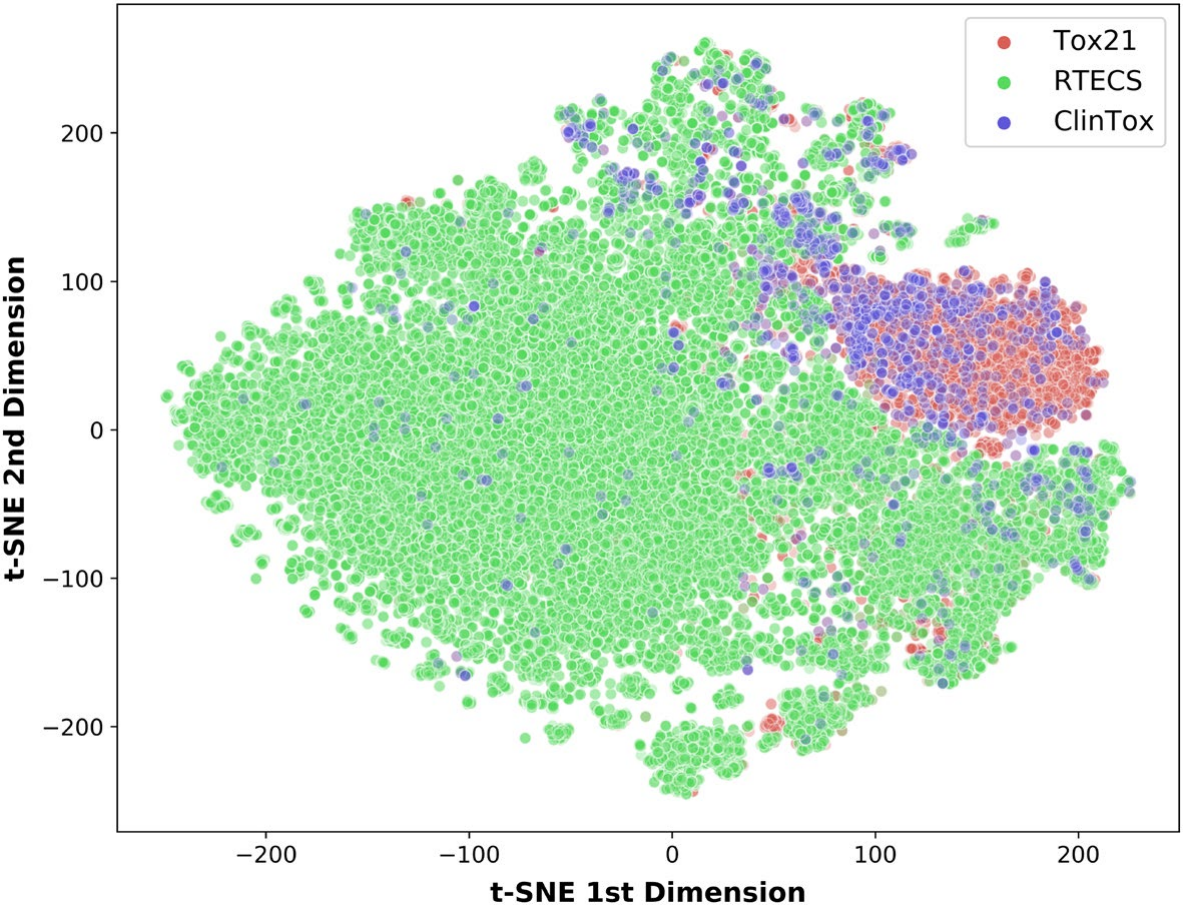
t-SNE of SMILES embeddings of chemicals in the Tox21, RTECS, and ClinTox datasets. Distances correlate to similarities of the chemicals across these datasets; shorter the linear distance, the more similar are the chemicals. ClinTox chemicals overlap more with Tox21 chemicals than with RTECS.

To investigate whether the type of chemicals within the in vitro, in vivo, and clinical datasets affected the ability of in vivo data to predict clinical toxicity, we visualized the relationships between the chemicals using t-distributed stochastic neighbor embeddings (t-SNE)^51^. t-SNE, a method that maps high-dimensional data to lower dimensions while preserving local similarities (i.e., distances between datapoints)^51^. We applied t-SNE mapping to SE of the chemicals in the Tox21, RTECS, and ClinTox datasets, with each dot representing a chemical and distance representing similarity (Fig. 5). The map is dominated by RTECS chemicals (green) due to the larger number of chemicals present in RTECS than both the Tox21 and ClinTox datasets. However, when examining overlap of the chemicals, the majority of the overlap is among ClinTox (purple) and Tox21 (red) chemicals, with some overlap between ClinTox (purple) and RTECS (green) chemicals. Thus, chemicals present in the clinical dataset (ClinTox) are more related to the chemicals present in the in vitro dataset (Tox21) rather than those in the in vivo (RTECS) dataset.

### Contrastive molecular-level explanations of toxicity

STDNN and MTDNN have improved the accuracy in predicting clinical toxicity. However, these DNNs cannot explain why a molecule was predicted to be toxic. To improve the trustworthiness of our results and to expand the current scope of explaining toxicity predictions, we adapted the contrastive explanation method (CEM)^43^ for molecular-level explanations of toxicity predictions. Specifically, we have adapted the CEM to explain in vitro, in vivo and clinical toxicity predictions, made by the STDNN trained on Morgan fingerprints (STDNN-FPs). STDNN-FPs were chosen as they can provide easy-to-understand substructure level explanations from their FP input, while maintaining consistent and significant accuracy across all platforms.

The CEM more comprehensively explains DNN predictions by identifying present (pertinent positive, PP) and absent (pertinent negative, PN) substructures within the molecules that correlate to a prediction. For instance, for a molecule predicted to be “toxic”, the PP substructures are the minimum and necessary substructures within the molecule that correlate to the “toxic” prediction. Conversely, the PN substructures represent the minimum and necessary substructures missing from the molecule that when added convert the “toxic” prediction to “nontoxic”. To this end, we obtained PP and PN substructures for all of the molecules in the ClinTox, Tox21 and RTECS test sets. These PP and PN substructures illuminate the decisions made by the STDNN-FPs and provide substructures that correlate to “toxic” and “nontoxic” predictions. We focused on only correct “toxic” and “nontoxic” predictions in the test set of ClinTox, Tox21, and RTECS. The CEM collected PP and PNs for correctly predicted chemicals in tests datasets of ClinTox, Tox21, and RTECS. For each molecule, up to the first ten explanations by weight were collected, totaling 166,308 obtained PP and PN substructures.

#### Most common PP and PN substructures for “toxic” and “nontoxic” molecules

**Figure 6:**
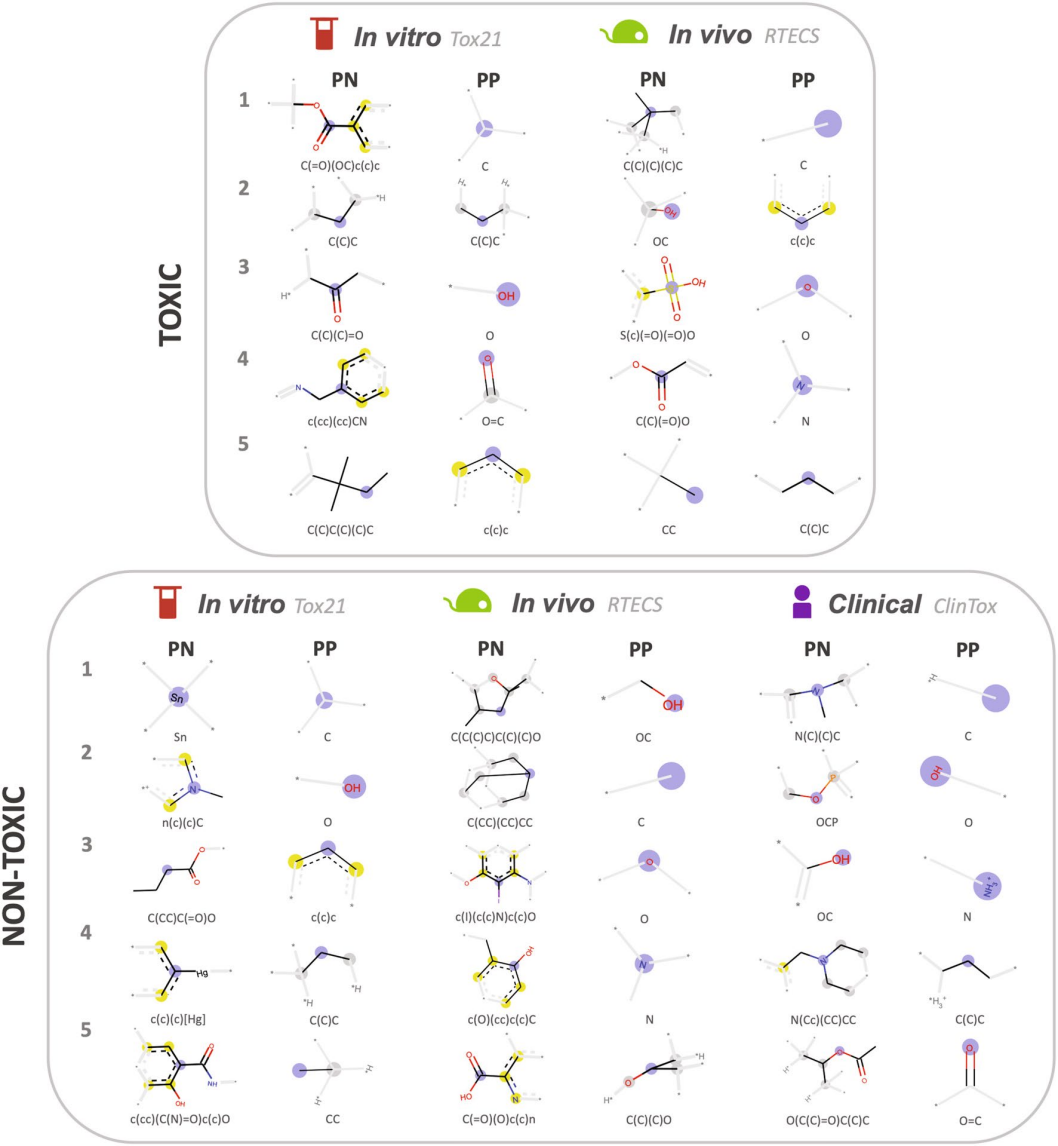
Most common PP and PN substructures of correctly predicted toxic and nontoxic molecules across the Tox21, RTECS, and ClinTox endpoints. ClinTox only had 1–2 examples of toxic molecules in the test set and thus was excluded here. All top 10 in Supplementary Figs. S7 and S8.

To focus on prevalent molecular features for explanations from the $\sim$ 170,000 substructures obtained, we analyzed the ten most common PP and PN substructures by frequency for correct “toxic” and “nontoxic” in vitro, in vivo and clinical predictions (top five are shown in Fig. 6, top ten in Supplementary Figs. S7+S8). Any matches in frequency were resolved by using the weight order. These top ten most common PP and PNs represent the ten most common explanations for “toxic” and “nontoxic” predictions. However, for the highly skewed ClinTox test set, with only 1–2 “toxic” molecules, only the “nontoxic” predictions were examined. Along with the substructures, the CEM also provides the most significant (central) atom of the substructure (blue circles in Fig. 6).

Two approaches were used to obtain explanations for “toxic” predictions of molecules by the STDNN: (1) PP substructures present in “toxic” molecules, and (2) PN substructures missing from “toxic” molecules which, if present, would change the prediction from “toxic” to “nontoxic” (Fig. 6 and Supplementary Fig. S7). From the top ten most frequent PPs of molecules predicted to be “toxic”, common substructures were identified for both Tox21 and RTECS, heavily involving nucleophilic N, O, and aryl groups (Fig. 6 and Supplementary Fig. S7). The most common was just a carbon fragment, perhaps due to the abundance of this substructure in the Morgan fingerprints. Substructures with oxygen were also common, either as a carbonyl (Tox21 and RTECS), ketone (Tox21), or as an ether (RTECS). Different portions of aromatic rings were identified multiple times, within an aryl ring (Tox21 and RTECS), or within a benzyl group (Tox21 and RTECS). Finally, N was obtained for both Tox21 and RTECS. Thus, the presence of nucleophilic N, O, and aryl containing substructures was commonly identified as explanations for in vitro and in vivo predictions of toxicity.

Explanations of “toxic” predictions obtained from the absence of PN substructures from molecules predicted to be toxic, most commonly contained carbon fragments and substructures with N and O for both Tox21 and RTECS, but also identified aryl halides for Tox21 and sulfur (S) containing substructures for RTECS (Fig. 6 and Supplementary Fig. S7). For O containing substructures, aromatic and aliphatic carboxylic (Tox21 and RTECS) moieties were identified. N containing substructures were present as an aromatic amine (Tox21), as the heteroatom in cephalosporins (RTECS), an imine (RTECS), or a nitrite (Tox21). For substructures that differed between Tox21 and RTECS, Tox21 specified an aryl chloride, while RTECS specified S within an aromatic sulfonic acid group.

Similarly, the ten most common explanations for molecules predicted to be “nontoxic” were obtained (Fig. 6, Supplementary Fig. S8). From the PP of molecules predicted to be “nontoxic”, common substructures were identified for ClinTox, Tox21, and RTECS to be aryl fragments, and small substructures with N and O (Fig. 6, Supplementary Fig. S8). Aryl fragments were present for Tox21 and ClinTox. Small substructures with O were frequent for all datasets, specifying only O, a hydroxyl, or a carbonyl. Substructures with only the N (Tox21, RTECS, ClinTox) or as an amide (RTECS) were also present.

Explanations of “nontoxic” predictions obtained from the absence of PN substructures from molecules predicted to be nontoxic contained various complex aromatic substructures with N, O, S, P, I and F, and heavy metals (Tin, Sn and Mercury, Hg) (Fig. 6 and Supplementary Fig. S8). The difference among Tox21, RTECS, and ClinTox was in the complexity of the aryl structures and type of heteroatom. ClinTox had the most complex aryl substructures with different combinations of N, O, P, or F, followed by RTECS with complex aryl substructures containing N, O, S, and the halide I. Finally, Tox21 had the least number of complex aromatic structures with N as a heterocyclic amine or an aromatic amide, with Hg as a substituent (organomercury), or with O as a phenol. N and O containing aliphatic substructures were also common through all the endpoints, while Tox21 also specified aliphatic substructures with Sn.

The importance of the top ten most frequent pertinent features extracted by the CEM was verified by a simple implementation of the Genetic Algorithm (GA)^52^. We matched the obtained PP and PN substructures to features selected by GA, a classical feature selection method^52^. The GA selects optimal input features for a prediction by an algorithm inspired by natural selection^52^. Features selected from the GA were also identified within the top ten most frequent pertinent substructures obtained by the CEM (Supplementary Figs. S9, S10). From the top ten explanations of “toxic” predictions, carbon fragments and aryl groups from both PP and PNs (Tox21, RTECS) matched with features obtained by the GA. Other matches to GA features within the top ten “toxic” explanations included ether (Tox21), ketones (Tox21), or amines (RTECS) as PPs, and aromatic amines (Tox21), or hydroxyl (RTECS) as PNs. GA features matched to the top ten pertinent substructures for “nontoxic” predictions as well (Supplementary Fig. S10). Matches to PPs contained aryl fragments (Tox21, ClinTox), O (Tox21), carbonyl (Tox21), ether (RTECS), hydroxyl (ClinTox, RTECS, Tox21), and amines (ClinTox). Complex aryl substructures with N, Hg (Tox21), O, P, F (ClinTox), and a phenol ether (RTECS) matched as PNs of nontoxic predictions. Thus, there is agreement seen between the features selected by the GA, and the top most frequent pertinent features extracted by the CEM. The advantage of examining CEM features over GA features, is the ability of the CEM to indicate the minimal necessity of a certain feature (substructure) for a specific “toxic”/“nontoxic” prediction by both the presence and absence. The GA simply gives a broad indication of near optimal input features for the entire model. Using a probabilistic stochastic search^52^, the GA may not return the same results each run even with the same parameters.

In the process of explaining “toxic” and “nontoxic” predictions, the CEM obtains PP and PN substructures correlating to toxicity and nontoxicity, i.e. computationally extracting toxicophores and nontoxic substructures. PP of “toxic” predictions and PN of “nontoxic” predictions are both substructures that correlate to toxic predictions, i.e., toxicophores. Conversely, PP of “nontoxic” predictions and PN of “toxic” predictions are substructures correlating to nontoxic predictions, i.e., nontoxic substructures. Thus, the above identified PP and PN substructures are also the top ten most commonly identified toxicophores and nontoxic substructures by the CEM within each dataset.

#### Verification of pertinent features extracted to known toxicophores

To verify the correlation between the obtained PP and PN substructures and the toxicity predictions, we matched the PP and PN substructures correlating to toxicity with known toxicophores. PP and PNs correlating to toxicity, or toxicophores, are obtained from the CEM by: (1) PPs of molecules correctly predicted to be “toxic”, and (2) PNs of molecules correctly predicted to be “nontoxic” that would flip the molecule to be classified as “toxic”.

**Figure 7:**
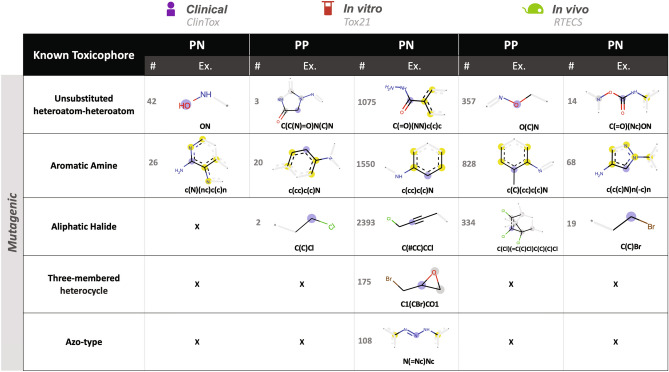
Matched toxicophores. Top three (ClinTox, RTECS) or top five (Tox21) matched known toxicophores to toxicophores collected from the CEM as PP of toxic molecules and PN of nontoxic molecules. For Tox21, the top five most frequent matches were examined due to the large number of matches. Three types of known toxicophores were matched: experimental and computational mutagenic toxicophores, and reactive substructures commonly used to filter molecules. The table provides the number (#) of matches, and specific examples (Ex.). Only mutagenic toxicophores are displayed in table, full list is given in Supplementary Fig. S11.

The literature contains a vast and diverse array of known toxicophores. Mutagenic toxicophores, in particular, have been widely used to verify results of computationally predicted toxicophores^8,53^. Here, we matched the toxicophores obtained from the CEM to known mutagenic toxicophores collected in vitro experimentally^54^ (Fig. 7), or computationally^53^ (Supplementary Fig. S11), and known reactive substructures commonly used to filter molecules^55^ (Supplementary Fig. S11). The CEM was able to identify toxicophores across all these types of known toxicophores, both from PP substructures of correctly predicted toxic molecules and PN substructures of correctly predicted nontoxic molecules.

Examining the top three (ClinTox, RTECS) or the top five (Tox21) most frequently matched toxicophores, the CEM identified known toxicophores that are common to all endpoints (purple in Fig. 7), to ClinTox and Tox21 (magenta in Fig. 7)), to Tox21 and RTECS (orange in Fig. 7), or unique to Tox21 (red in Fig. 7)) or to RTECS (green in Fig. 7)). For Tox21, top five most frequent matches were examined due to the large number of matches. Both Tox21 and RTECS recovered toxicophores common with ClinTox (unsubstituted heteroatom-heteroatoms, aromatic amines, and Michael receptors); however, only Tox21 identified thioesters, a reactive substructure present in ClinTox. Aromatic nitro, aliphatic halide, alkyl halide, heteroatom-heteroatom single-bond substructures were matched toxicophores found in both Tox21 and RTECS, but not in ClinTox. Uniquely, PPs and PNs in Tox21 matched to reactive cyanide, three-membered heterocycle, azo-type, carbonyl/ether, epoxides, thioepoxides, and disulfide substructures, while only RTECS matched to 1,2-dicarbonyls within the top three to five matched toxicophores. Notably, a larger number of toxicophore matches were found in vitro (in the 1000 s) or in vivo (in the 100 s), compared to the clinical endpoint (in the 10 s).

Thus far, we have matched known toxicophores to only PP and PNs correlating to toxicity. To discern whether the CEM correctly pinpoints PP and PNs substructures correlating to toxicity, we further matched all collected PP and PNs to known toxicophores. We expect to see a larger number of matches with PP and PN substructures correlating to toxicity (toxicophores), than to the converse (nontoxic substructures). Indeed, for ClinTox and Tox21, but not for RTECS, there are a larger number of matches to known toxicophores with CEM-derived toxicophores than with CEM-derived nontoxic substructures (Supplementary Fig. S12).

## Discussion

We have demonstrated an improvement in predicting clinical toxicity using pre-trained SMILES embeddings as input molecular representations within a multi-task deep neural network that simultaneously learns in vitro, in vivo, and clinical toxicity tasks. Through our multi-task model, we investigated the benefits of learning from more diverse toxicity data in in vitro, in vivo, and clinical platforms for predicting clinical toxicity. We also leveraged pre-trained molecular SMILES embeddings as inputs, which better captured intermolecular relationships within a larger corpus. Compared to the existing MoleculeNet baseline and our single-task models, both the multi-task setting and SMILES embeddings contribute to the marked improvement in AUC-ROC on the clinical platform while providing comparable performance on the in vitro platform and no improvement on the in vivo platform (Fig. 2, “Deep single-task and multi-task predictive models: Morgan fingerprints and SMILES embeddings”). Regarding the balanced accuracy, the multi-task model improved on the clinical platform and showed no improvement on the *in vitro* or *in vivo* platforms (Fig. 3,  “Deep single-task and multi-task predictive models: Morgan fingerprints and SMILES embeddings”).

The improvement in AUC-ROC correlates with the size of the datasets. ClinTox is the smallest dataset ($\sim$ 1000 compounds), followed by Tox21 ($\sim$ 8000 compounds) and RTECS ($\sim$ 40,000 compounds). It is possible that leveraging the relationships among chemicals from a large corpus and among in vitro, in vivo and clinical tasks improves learning on smaller datasets but not on larger datasets. Additionally, the poor performance in RTECS could result from the distinct nature of chemicals within RTECS, as displayed by the lack of overlap of RTECS chemicals with ClinTox or Tox21 chemicals in the t-SNE mapping (“Testing the relative importance of in vivo data to predict clinical toxicity”, Fig. 5). A multi-task setting does not appear to help with a larger dataset of dissimilar chemicals but does help for the smaller clinical toxicity dataset.

The top-performing baseline model from MoleculeNet benchmark for each platform (Weave on ClinTox, GC on Tox21, IRV on RTECS) either considered graph neural nets or an ensemble of models. These MoleculeNet models were optimized via additional hyperparameter tuning. In contrast, without any additional hyperparameter tuning, leveraging bond connectivity information, or ensemble modeling, our multi-task deep predictive model provided reasonable accuracy across all platforms, even on RTECS (AUC-ROC of $0.78 \pm 0.02$ with SMILES embeddings).

The ability to predict accurately and consistently across the in vitro, in vivo, and clinical platforms is important. Currently, predictive toxicology models primarily focus on predicting within one platform (Fig. S1 in Supplementary). Even though these toxicities are measured at differing granularities, the relationships among these different platforms might not be apparent if modeled separately, e.g., performance on the ClinTox task improved using the multi-task model (MTDNN-SE) as compared to the single-task models (STDNN-FP and STDNN-FE) (Fig. 2, “Deep single-task and multi-task predictive models: Morgan fingerprints and SMILES embeddings”). A multi-task model can overcome the small overlap in common chemicals across these platforms by sharing weights while training. For instance, even with the small overlap of the RTECS dataset with ClinTox or Tox21, the MTDNN-SE could still reasonably predict the RTECS endpoints. Predicting across these platforms together also provides a methodology to test the ability of a particular type of platform to predict clinical toxicity.

Using our multi-task model and its transfer learning counterpart, we demonstrated the minimal relative importance of in vivo data to make accurate predictions of clinical toxicity. The addition of in vivo data in the MTDNN or its transfer learning counterpart did not improve clinical toxicity (“Testing the relative importance of in vivo data to predict clinical toxicity”). Instead, the addition of in vitro data to clinical data was sufficient in improving the predictions of clinical toxicity by AUC-ROC (Fig. 2, “Deep single-task and multi-task predictive models: Morgan fingerprints and SMILES embeddings”). In vivo data only helped increase balanced accuracy on the clinical task when the MTDNN was trained on Morgan fingerprints as input (light red versus light purple or light orange in Fig. 3), but not when trained on SMILES embeddings (dark purple versus dark red or dark orange in Fig. 3). Thus with the use of SMILES embeddings as input, in vivo data is not needed for high AUC-ROC and balanced accuracy performance on the clinical task. Moreover, the chemicals in the clinical dataset (ClinTox) were more similar to chemicals in the in vitro dataset (Tox21) than in the in vivo dataset (RTECS), as examined through the t-SNE mappings (Fig. 5). The present study examines the ability of acute oral toxicity data in mice as in vivo data, and nuclear receptor and stress response assays as in vitro data, to predict the failure of drugs in clinical phase trials in humans. Broader in vivo and in vitro datasets could augment our conclusions and will be investigated in the future.

We have improved accuracy in predicting clinical toxicity using DNN models, but with the caveat of reduced explainability in the models which we tackled in this study^56^. DNNs are known for being “black-box” models due to their inability to describe why a prediction was made^57^. Explainability of models can improve trust and adoption of models into the healthcare industry^57^. Traditionally, explanations of toxicity predictions have been limited to pinpointing the presence of substructures (Section 1.1. of Supplementary). Recent work provides molecular counterfactual explanations on toxicity^58^ and other molecular property predictions^59^, using model agnostic^59^ or reinforcement learning-based deep graph explainers^58^. Contrastive Explanations, provide both present and absent substructures correlating to a prediction, and to our knowledge has only been applied by our previous work on one specific in vitro endpoint^46^. This current work expands on current molecular explanations by providing more complete, human-understandable, contrastive explanations on toxicity predictions across in vitro, in vivo, and clinical platforms, with both substructures that are absent and present in the chemicals. For this purpose, we explain predictions made by the STDNN-FP model. We chose this model because of its consistent performance across all tasks, agreeing with an earlier observation that simple descriptor-based (e.g., FP) models can provide better performance and higher computational efficiency than more complex models such as the graph-based ones^60^. We explained toxicity predictions across in vitro (Tox21), in vivo (RTECS), and clinical (ClinTox) tasks. The PP and PN substructures obtained by the CEM not only provided explanations on toxicity predictions, but also suggested possible toxicophores and nontoxic substructures. The uniqueness of this approach is the computational identification of toxicophores from substructures absent from a molecule that would flip the prediction from nontoxic to toxic. We have thus, in the process of explaining DNN toxicity predictions, created a new and more comprehensive approach of obtaining computational toxicophores and nontoxic substructures.

Common toxicophores were obtained across all platforms, both by PPs of “toxic” predicted molecules and PNs of “nontoxic” predicted molecules, containing O and N groups, P, S, I and F, or aryl substructures of varying complexity with the most complex substructures present in ClinTox (“Contrastive molecular-level explanations of toxicity”, Fig. 6). Similar nontoxic substructures were also identified across all platforms by PP substructures of nontoxic molecules and PNs of toxic molecules, containing aryl groups, and smaller O- and N-containing substructures. The mismatch was in PNs of toxic molecules, with RTECS containing S substructures (i.e., sulfonic acid) and Tox21 containing aryl chlorides (Fig. 6). These toxicophores identified from the CEM were verified by matching to known toxicophores, both by PP substructures present in toxic molecules and PNs substructures absent in nontoxic molecules (“Contrastive molecular-level explanations of toxicity”, Fig. 7).

The CEM-derived and verified toxicophores for the clinical task were found for both the in vitro and in vivo tasks, supporting the validity of initial virtual screening for known in vitro and in vivo toxicophores. A larger number of verified toxicophores was found for the in vitro (Tox21) task ($\sim$ 1000 s), followed by the in vivo (RTECS) task ($\sim$ 100 s), and the last by the clinical task ($\sim$ 10 s), perhaps due to the known toxicophores primarily being collected from other in vitro experiments, as well as due to difference in dataset size. The more extensive toxicophore recovery from in vitro and in vivo endpoints potentially uncovers a preference in toxicophore data towards in vitro and in vivo experimental data.

The relevance to the computationally obtained toxicophores and nontoxic substructures from CEM explanations beyond the three datasets (Tox21, ClinTox, RTECS acute oral toxicity) used here will be explored in future work. Also, the model trained here can also be further fine-tuned on additional labeled datasets not covered in this study to cover a larger set of chemicals. Applicability domain (AD) can establish the appropriateness of applying our current model to new chemicals^61^. Though a variety of methods can determine AD^61^, estimating the uncertainty of prediction probabilities from an ensemble of deep models is an easy to implement non-Bayesian solution^62,63^. To determine whether a new chemical is within the chemical landscape of our model for a specific task, the uncertainty of the predictive probability of the new chemical should be less than the largest uncertainty found on training chemicals^61^ (Supplementary Fig. S13). The uncertainty was estimated by the maximum variance in predictive probabilities from five randomly initialized MTDNN-FPs^62^ trained on the three platforms (Tox21, RTECS, ClinTox) (Supplementary Fig. S13). The provided uncertainties demonstrate the procedure to obtain the applicability domain of the MTDNN-FP model trained on all three datasets, but can be expanded to any of our models.

The obtained toxicophores are thus most relevant to the studied endpoints within these small molecule datasets, i.e., to the nuclear receptor and stress response assays (Tox21), to the acute oral toxicity in mice (RTECS), and to the toxicity in clinical phase trials (ClinTox). Though toxicophores are used across different endpoints, their usage remains restricted to the initial screening for potentially toxic molecules^55^. It is important to note that our toxicity explanations are also an initial approach to provide more confidence and interpretation in toxicity predictions made from DL models, which can help in initial screening for toxic molecules, including drug candidates. The presence or absence of a toxicophore, physiologically, does not necessarily guarantee a molecule will be toxic; as potential biological targets, pathways and interactions are not taken into account in this analysis. Another limitation of our approach is only the identification of missing substructures (PNs) and not their relative location on the compound. Often carbon fragments were identified as both PP and PN substructures, due to their prevalence in Morgan Fingerprints, however more meaningful substructures were also pinpointed.

The CEM is focused on explaining neural network decisions, which is a good starting point to illuminate “black-box” DNN-based models that have increasingly been applied to the chemical and drug toxicity predicitions while also providing a list of potential toxicophores and non-toxicophores. Future work will address comparison of explanations resulting from different predictive models with varying architectures and input modality, as well as of different post-hoc interpretability methods. We hope studies like ours can help chart a path toward optimizing the number of future toxicity screen experiments across different in vivo, in vitro and clinical endpoints.

## Conclusion

We have demonstrated the advantage of employing a deep neural net to predict toxicity across in vitro, in vivo, and clinical platforms. With pre-trained molecular SMILES embeddings as input, the multi-task model yielded improved or on par clinical toxicity predictions to current baseline and state-of-art molecular graph-based models. Unlike graph neural nets, our framework takes advantage of reduced inference costs from language models when using pre-trained SMILES embeddings. The results presented here strongly suggest that there is a minimal relative importance of in vivo data for predicting clinical toxicity in particular when unsupervised pre-trained SMILES embeddings were used as an input to multi-task models; thus, providing possible guidance on what aspects of animal data need not be considered in predicting clinical toxicity. We further provided a more complete and consistent molecular explanation of the predicted toxicities of a performant deep neural net across different platforms by analyzing the contrastive substructures present within a molecule. To our knowledge, this is the first work to explain with both present and absent substructures predictions of clinical and in vivo toxicity. Thus we have created a framework to provide improved and explainable clinical toxicity predictions, while limiting the amount of animal data used.

## Methods

### Deep single-task and multi-task predictive models

#### Input molecular representations

Two types of computable molecular representations of chemical structures were used as input to the multi-task and single-task deep predictive models: Morgan fingerprints (FP), and pre-trained SMILES embeddings (SE). FPs, simpler and more widely used^44^, represent molecules as a vector indicating presence of a circular substructure within varying radii around an atom. FPs were calculated by RDKit^64^, with a Morgan radius of 2 and bit size of 4096. SE were created using a neural network-based translation model that translates from non-canonical SMILES to canonical SMILES, similar to the model proposed by Winter et al.^65^. The neural network is a bidirectional Gated Recurrent Unit (GRU) with three encoder and decoder layers an embedding size of 128. The encoder layers have a dimension of 512 units and the decoder layers have a dimension of 256 units. Further, in order to learn a better representation, we jointly train a regression model that can predict molecular properties that can be computed from the molecular structure. The properties predicted by the regression model are: logP, molar refractivity, number of valence electrons, number of hydrogen bond donors and acceptors, Balaban’s J value, topological polar surface area, drug likeliness (QED) and Synthetic Accessibility (SA). This model was trained on 103 million chemicals in PubChem^66^ and 35 million chemicals in ZINC12^67^. The trained translation model was then applied to the chemicals present in the in vitro, in vivo, and clinical datasets to obtain their embeddings.

#### Datasets: in vitro, in vivo, clinical

The single-task and multi-task models predict binary classes, whether a chemical is toxic or not, for each of the in vitro, in vivo, and clinical platforms. Twelve binary classes were defined for the in vitro platform, collected from the Tox21 challenge, a subset of the broader “Toxicology in the 21st Century” initiative that experimentally tests in vitro the ability of a large number of chemicals to disrupt biological pathways through high-throughput screening (HTS) techniques^17–19^. In particular, twelve in vitro assay results were provided in Tox21, testing seven different nuclear receptor signaling effects, and five stress response effects^16^ of 8014 molecules in cells^45^.

One binary class was defined for the clinical platform from the ClinTox dataset^45^, as whether a chemical was approved or failed due to toxicity in clinical phase trials. ClinTox is a curated dataset by MoleculeNet^45^, a benchmark for molecular machine learning models specifying datasets, models and evaluation criteria. ClinTox contains 1491 drugs that have either been approved by the FDA (collected from the SWEETLEAD database^68^) or failed clinical trials as reported by the Aggregate Analysis of ClinicalTrials.gov (AACT) dataset^69^.

One in vivo class was parsed from the commercially available RTECS (Registry of Toxic Effects of Chemical Substances) dataset. This dataset contains in vivo toxicity data curated from literature across various endpoints (acute, mutation, reproductive, irritation, tumorigenic, multiple-dose toxicities) in the form of different toxic measurements. We focused on acute oral toxicity in mice due to the largest number of examples. For 42,639 chemicals, binary class was defined by LD_50_ (lethal dose for 50 percent of the population) cutoff of 5000 mg/kg ($\le$ as toxic, > as nontoxic) as specified by EPA (Environmental Protection Agency) and GHS (The Globally Harmonized System of Classification and Labeling of Chemicals).

#### Multi-task and single-task DNN architecture

The Multi-task Deep Neural Network (MTDNN) consists of an input layer of either Morgan fingerprints (radius = 2) or SMILES embeddings, passed to two layers (2048, 1024 nodes) shared by all tasks, and further two layers (512, 256 nodes) for each separate task. The output layer (one node) corresponds to toxic/nontoxic labels for each endpoint and is activated by a sigmoid (Fig. S14 in Supplementary). The datasets are split into training/validation/test sets of 0.8/0.1/0.1. The model is trained on random batches of 512 per training step. The number of epochs is set at the lowest validation loss (one to four epochs depending on the seed). Binary cross entropy was chosen as the loss function, and it was optimized using the Adam optimizer with a learning rate of 0.001.

The Single-task Deep Neural Network (STDNN) consists of two layers (512, 256 nodes) and one output layer (one node) activated by a sigmoid. For tasks with multiple classes, the average of the area under the receiver operating characteristic curve (AUC-ROC) and balanced accuracy was taken. The same data splits, and training hyperparameters are used as the MTDNN. The STDNN and MTDNN models were run with NVIDIA k80 GPUs on the Cognitive Computing Clusters at IBM.

To obtain baseline results from MoleculeNet on RTECS, the provided benchmark methods in the DeepChem^70^ MoleculeNet GitHub (https://github.com/deepchem/deepchem/tree/master/deepchem/molnet) was adapted to train and test the same models run on ClinTox and Tox21^34^, on RTECS. The MoleculeNet models tested were: Weave, GC, Bypass, Multitask, IRV, RF, XGBoost, KernelSVM, and Logreg. Wu et al.^34^ provide details on these models.

AUC-ROC and balanced accuracy were used as metrics of performance for the models. Balanced accuracy is the average of sensitivity and specificity. The former is the fraction of correctly classified positive classes out of all possible positives in the dataset, i.e., fraction of true positives out of correctly classified positives and falsely classified negatives, such that,

1 $$\begin{aligned} \begin{aligned} Sensitivity = \frac{TP}{TP + FN}. \end{aligned} \end{aligned}$$

Conversely, the specificity is this measure for the true negatives of the model, i.e. the fraction of true negatives correctly classified out of the total number of negatives in the dataset (both the correctly classified negatives and the falsely classified positives), such that,

2 $$\begin{aligned} \begin{aligned} Specificity = \frac{TN}{TN + FP}. \end{aligned} \end{aligned}$$

Thus, balanced accuracy takes into account both the true negative and true positive distribution in the overall performance of model, such that,

3 $$\begin{aligned} \begin{aligned} Balanced\ Accuracy = \frac{Sensitivity + Specificity}{2}. \end{aligned} \end{aligned}$$

The graphs for AUC-ROC (Fig. 2) and balanced accuracy (Fig. 3) were obtained from R^71^. The legend and labels were formatted on PowerPoint, and the final figures were converted to .eps file on Adobe Illustrator^72^.

### Transfer learning

The base model was trained with the multi-task DNN on different combinations of in vitro (Tox21) and in vivo (RTECS) tasks for 2–8 epochs depending on the lowest validation loss. From the base multi-task DNN model, the two layers shared among the tasks were extracted, and their weights frozen. To this, two additional layers were added for the ClinTox task on which transfer learning was needed. The number of epochs on this additional training with the transfer learning task was varied; one epoch was chosen to limit the training on the transferred task. The same ClinTox, Tox21, and RTECS testing data as the MTDNN were used to evaluate the models.

### t-Distributed stochastic neighbor embedding

t-Distributed stochastic neighbor embedding or t-SNE^73^ visualizes high-dimensional data by mapping them to a low-dimensional space indicating the similarities between different points. We visualized SMILES embeddings of the chemicals present in the Tox21, RTECS, and ClinTox datasets through a t-SNE set at 40 perplexity, 5000 iterations, and 1000 learning rates. sklearn.manifold.TSNE method in the sckit-learn package was used^74^. The t-SNE figure (Fig. 5) was graphed on Matplotlib^75^.

### Genetic algorithm for feature selection

Genetic algorithm (GA) searches for optimal features for a prediction with a process adopting natural selection^52^. A simple implementation of the GA was used to select features (substructures) for prediction of “toxic”/“nontoxic” for each of the endpoints. The input used were FPs with the same training set as the single-task models. The GA identifies bits within the FP that are the most optimal for a toxicity prediction. The bits match to a specific substructure. Matches to the pertinent substructures obtained by the CEM was done by matching bits.

The sklearn-genetic package in the sckit-learn^74^ was used for the GA. Using the GeneticSelectionCV method in sklearn-genetic package^74^, simple single-task Random Forest classifiers were used as estimators, with five-fold cross validation, scoring with accuracy, crossover independent probability of 0.5, mutation independent probability of 0.04. Number of generations was 50 for Tox21 and ClinTox, and 45 for RTECS. Maximum features was set to 1000 for ClinTox, 100 for Tox21, and 300 for RTECS. Size of population was selected as 300 for ClinTox, and 100 for Tox21 and RTECS. The full selected features from the GA are given in the supplied code.

### Applicability domain

Applicability domain was defined by uncertainty in predictive probabilities calculated over five ensembles^61,62^ of the MTDNN-FP trained on all three platforms (Tox21, ClinTox, RTECS). Seeds of 122, 123, 124, 125 and 126 were used to randomly initialize each MTDNN-FP. The architecture, loss, and hyperparameters were kept the same as our defined MTDNN-FP. The datasets were split into training/validation/test sets of 0.8/0.1/0.1. The variance in predictive probability on the training chemicals over the five models was calculated as an estimate of the uncertainty of the model^62^. A new chemical falls within the AD of the model if the variance of its predictive probability is less than the maximum variance found for each task for a given model^61^. We have provided the maximum variance for the MTDNN-FP trained on all three platforms (Supplementary Fig. S13).

### Contrastive explanations method

The contrastive explanations method^43^ provides explanations to rationalize the classification of an input by identifying the minimal and necessary features which are both present and absent for a particular classification. The CEM thus introduces the notion of Pertinent Negative (PN) and Pertinent Positive (PP). A PN is a subset of the feature set necessary for a classifier to predict a given class, while a PP is the minimal subset of features whose presence gives rise to its prediction.

The CEM obtains the PN and PP via an optimization problem to look for a required minimum perturbation to the model, using a projected fast iterative shrinkage-thresholding algorithm (FISTA)^43^. A given input example $(x_0, t_0)$ is perturbed, with $x_0$ belonging to data space $\chi$ $(x_0 \in \chi )$ and its class label $t_0$ predicted from a neural network model. The perturbed example $(x \in \chi )$ is given by $x = x_o + \delta$, with $\delta$ defining the perturbation. The PP and PNs are obtained by optimizing on this $\delta$ perturbation. Further, an autoencoder $AE(\cdot )$ is used to assure closeness of the perturbed example *x* to the original example $x_0$, with *AE*(*x*) defined at the autoencoder reconstructed example *x*. The optimization problem for obtaining the PN of $(x_0, t_0)$ is given by,

4 $$\begin{aligned} \begin{aligned}{}&\underset{\delta \in \chi \mathbin {/} x_0 }{\text {min}}{} & {} c \cdot f_\kappa ^{neg}(x_o, \delta ) + \beta \left\| \delta \right\| _1 + \left\| \delta \right\| _2^2 + \gamma \left\| x_0 + \delta - AE(x_0 + \delta )\right\| _2^2. \end{aligned} \end{aligned}$$

While the optimization problem for obtaining PP for the given example is,

5 $$\begin{aligned} \begin{aligned}{}&\underset{\delta \in \chi \cap x_0 }{\text {min}}{} & {} c \cdot f_\kappa ^{pos}(x_o, \delta ) + \beta \left\| \delta \right\| _1 + \left\| \delta \right\| _2^2 + \gamma \left\| \delta - AE(\delta )\right\| _2^2. \end{aligned} \end{aligned}$$

Here, $\kappa$ defines the minimum confidence gap between the changed class probability and the original class probability, $\beta$ controls the sparsity of the solution, and $\gamma$ controls the degree of adherence to an additional autoencoder. Refer to Dhurandhar et al.^43^ for more details. Code is provided at https://github.com/IBM/Contrastive-Explanation-Method.

For Tox21, ClinTox and RTECS, $\kappa$ of 0.01 and $\beta$ of 0.99 was used for PNs, while $\kappa$ was set at 0.01 for PPs. $\beta$ for PPs was set at the minimum possible to obtain a substructure: 0.1 for ClinTox and RTECS, and 0.31 for Tox21. $\gamma$ was 0 for both PPs and PNs as an additional adherence to an autoencoder was not used. Maximum of 1000 iterations were allowed, with an initial coefficient of 10 used for the main loss term and permitting nine updates to this coefficient. The seed was set at 122.

As verification, toxicophores obtained by the CEM were matched to known toxicophores. Known toxicophores were curated as known mutagenic toxicophores from in vitro data^54^ and computational models^53^, or as commonly filtered reactive substructures^55^.

## Supplementary Information

Supplementary Information.

## Data Availability

The full Tox21 (https://github.com/deepchem/deepchem/tree/master/datasets) and ClinTox (https://github.com/deepchem/deepchem/tree/master/examples/clintox/datasets) datasets are publicly available. The RTECS dataset is commercially available from BIOVIA for a fee or by way of subscription, and cannot be shared.

## Code availability

We provide the code and the splits used for Tox21 and ClinTox datasets (https://github.com/IBM/multitask-toxicity).
