# Supplementary material for: Accurate clinical toxicity prediction using multi-task deep neural nets and contrastive molecular explanations
Source: Sci Rep. 2023 Mar 25;13:4908. doi: 10.1038/s41598-023-31169-8 (PMC10039880; doi:10.1038/s41598-023-31169-8)
Supplement: Supplementary file 1 — Supplementary Information. [file 41598_2023_31169_MOESM1_ESM.pdf]

Accurate Clinical Toxicity Prediction using Multi-task Deep Neural Nets and Contrastive Molecular Explanations

Bhanushee Sharma, Vijil Chenthamarakshan, Amit Dhurandhar, Shiranee Pereira, James A. Hendler, Jonathan S. Dordick, and Payel Das

Supplementary

1 Related Work

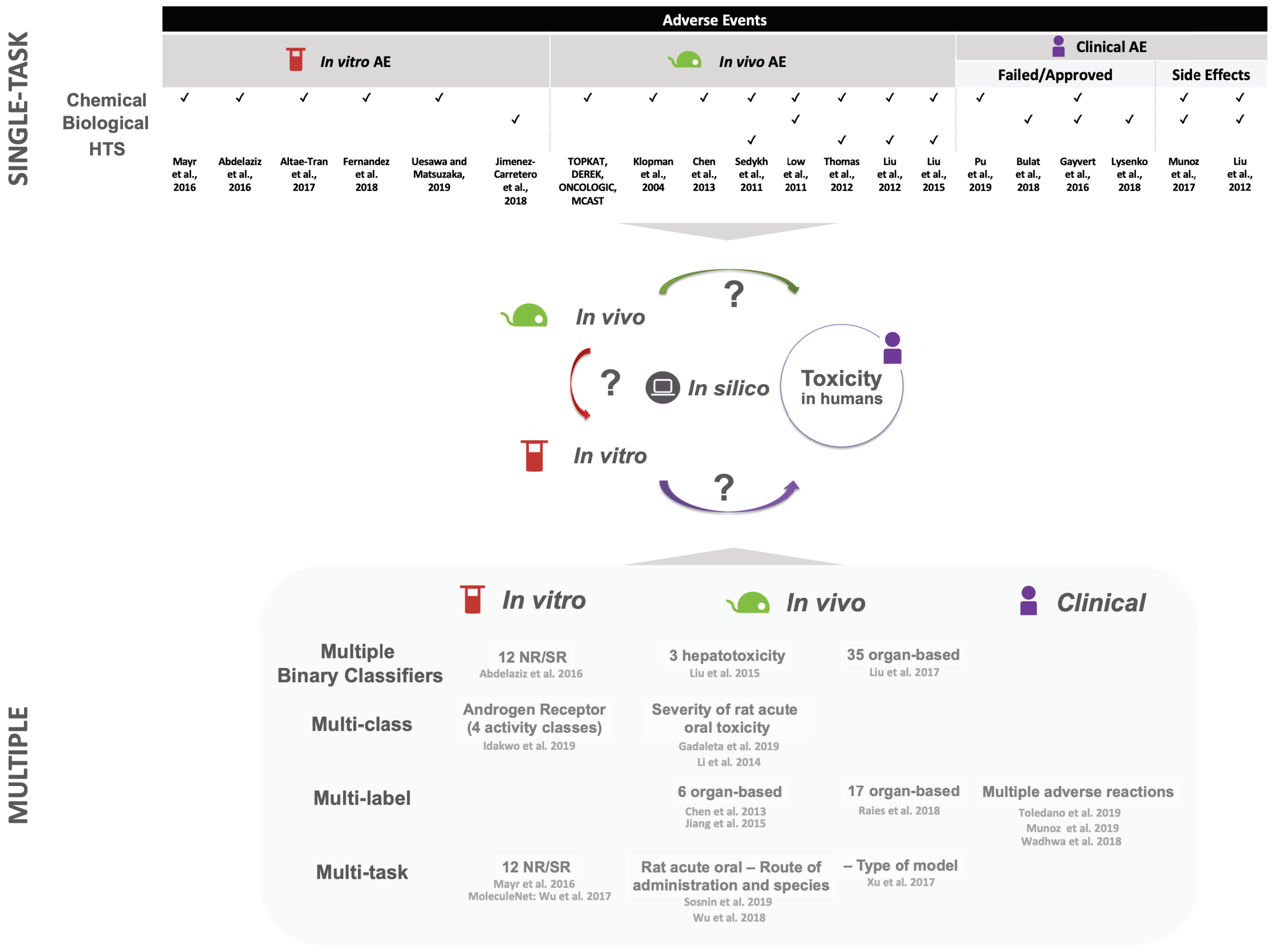

Figure S1. Problems in concordance and variety of input data used to predict a broad spectrum of endpoints across the *in vitro*, *in vivo* and clinical platforms with both single-task and multi-task models. Multi-class models [1–3] have predicted multiple toxic endpoints mutually exclusively per chemical. Multi-label models [4–6] have further generalized allowing mutually inclusive prediction of multiple endpoints for each chemical. Multi-task models [7–9] have learned from multiple tasks simultaneously and predict multiple or single endpoints across different tasks. The multi-label and multi-task models applied thus far are specific to tested platform(*in vivo*, *in vitro* or clinically). References: Liu et al. 2015 [10], Liu et al. 2017 [11], Gadaleta et al. 2019 [1], Li et al. 2014 [2], Chen et al. 2013 [4], Jiang et al. 2015 [5], Raies et al. 2018 [6], Sosnin et al. 2019 [8], Xu et al. 2017 [9], Wu et al. 2018 [7], Abdelaziz et al. 2016 [12], Idakwo et al. 2019 [3], Mayr et al. 2016 [13], Wu et al. 2017 [14], Toledano et al. 2019 [15], Munoz et al. 2019 [16], Wadhwa et al. 2018 [17]

1.1 Related Work on Molecular Toxicity Explanation

Explainable AI (XAI) in the Drug Discovery field is constantly expanding, particularly for molecular predictions [18]. Traditionally the focus has been on interpreting QSAR (Quantitative Structure-Activity Relationship) models [19], e.g., by compositional elemental/fragment or statistical analysis. Sharma et al. [20] explained differences between predicted toxic and nontoxic chemicals elementally, while Rasmussen et al. [21] and Polishchuk et al. [22] developed methods to examine fragment contributions to various molecular predictions. Sharma et al. also statistically determined significantly discriminating input features for toxicity predictions, such as solubility of chemicals [20].

Deep learning models for Drug Discovery applications have been explained by feature attribution, instance-based, graph convolutional based, self-explaining and uncertainty estimation methods [18]. Here we will discuss methods used to explain molecular predictions. Feature attribution methods explain by placing relevance on input features to a given prediction [18]. Within feature attribution methods, gradient-based and surrogate models have been used for molecular predictions. Gradient-based methods use the gradient of the model to determine feature importance, such as toxicophores being extracted from the DNN layers of DeepTox for *in vitro* toxicity predictions [13]. Graph-based neural nets have also been explained by gradient-based feature attribution methods, for *in vitro* toxicity predictions [23, 24] and for other molecular predictions [25]. Surrogate feature attribution models create a surrogate interpretable model which approximates the original model. Ramsundar et al. [26] applied the surrogate model, LIME (local interpretable model-agnostic explanations) [27], to extract toxicophores correlating to *in vitro* toxicity predictions. An extension to LIME, SHAP (Shapley additive explanations) [28], was applied to locate relevant substructures to compound activity predictions [29] or to rank different molecular descriptors [30]. Graph convolutional based XAI methods explain by identifying subgraphs within a molecular graph input correlating to a prediction. GNNExplainer, a model agnostic method extracted subgraphs (or toxicophores) for a specific *in vitro* toxic prediction that agreed with known mutagenic substructures [31]. Using the filters within a graph convolutional model, toxicophores were also extracted to explain *in vitro* toxicity predictions [32].

2 Full AUC-ROC comparison with baseline MoleculeNet

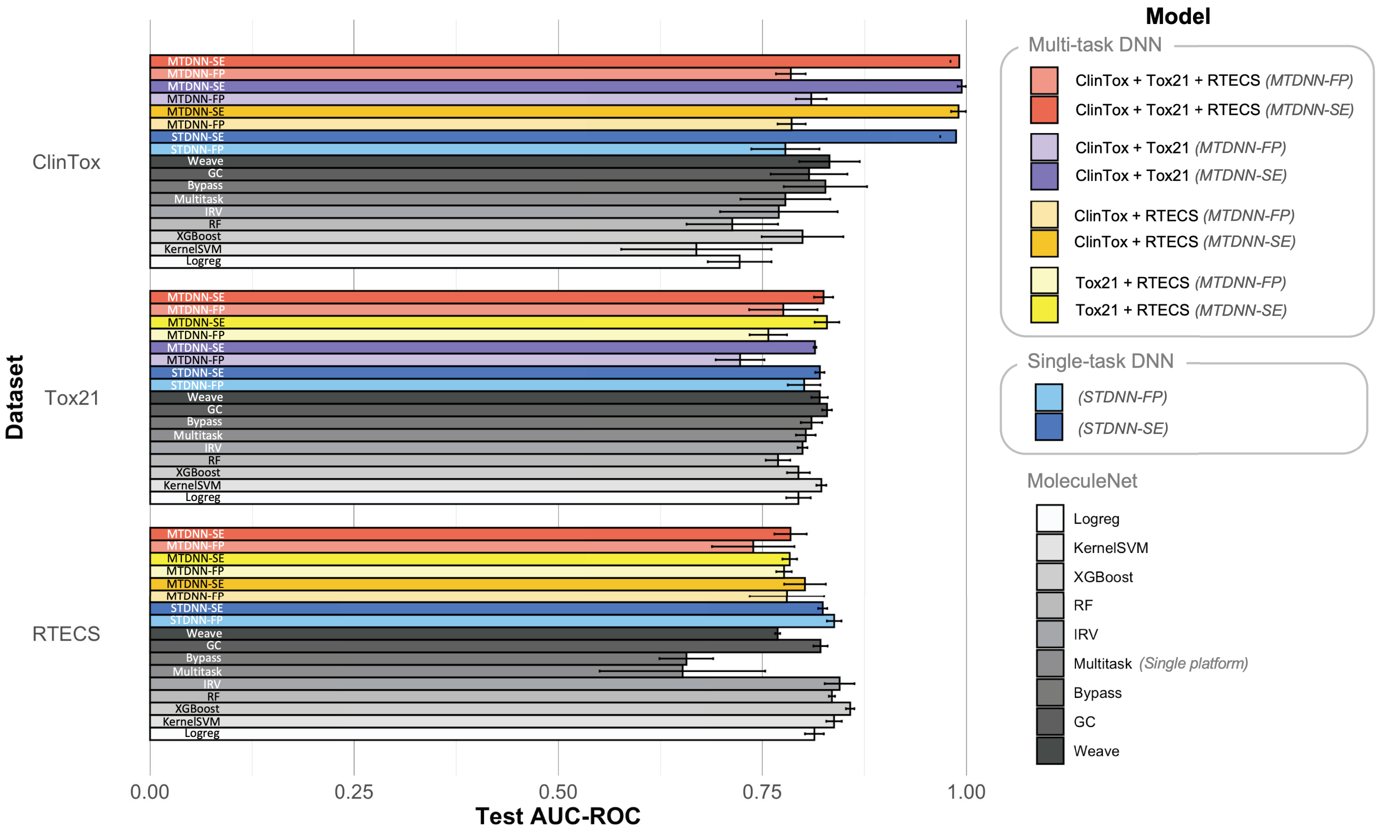

Figure S2. Test AUC-ROC values for ClinTox, Tox21, and RTECS predictions, comparing multi-task models to single-task and baseline MoleculeNet models, with SMILES embeddings and Morgan fingerprints as inputs.

3 Label Distribution

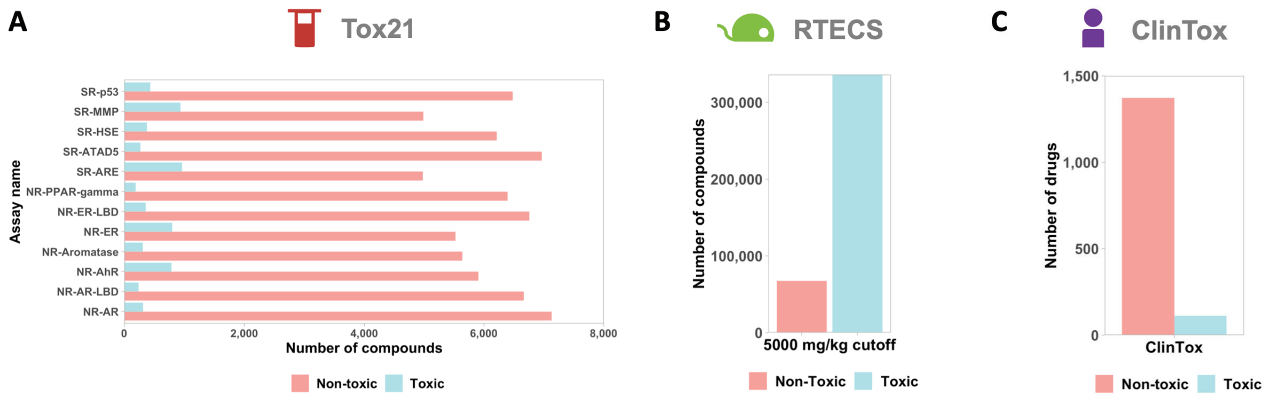

Figure S3. **Label distribution across *in vitro*, *in vivo* and clinical platforms.** Toxic and nontoxic label distribution for endpoints in (A) Tox21 (*in vitro*), (B) RTECS (*in vivo*), and (C) ClinTox (clinical).

4 Confusion matrices comparison

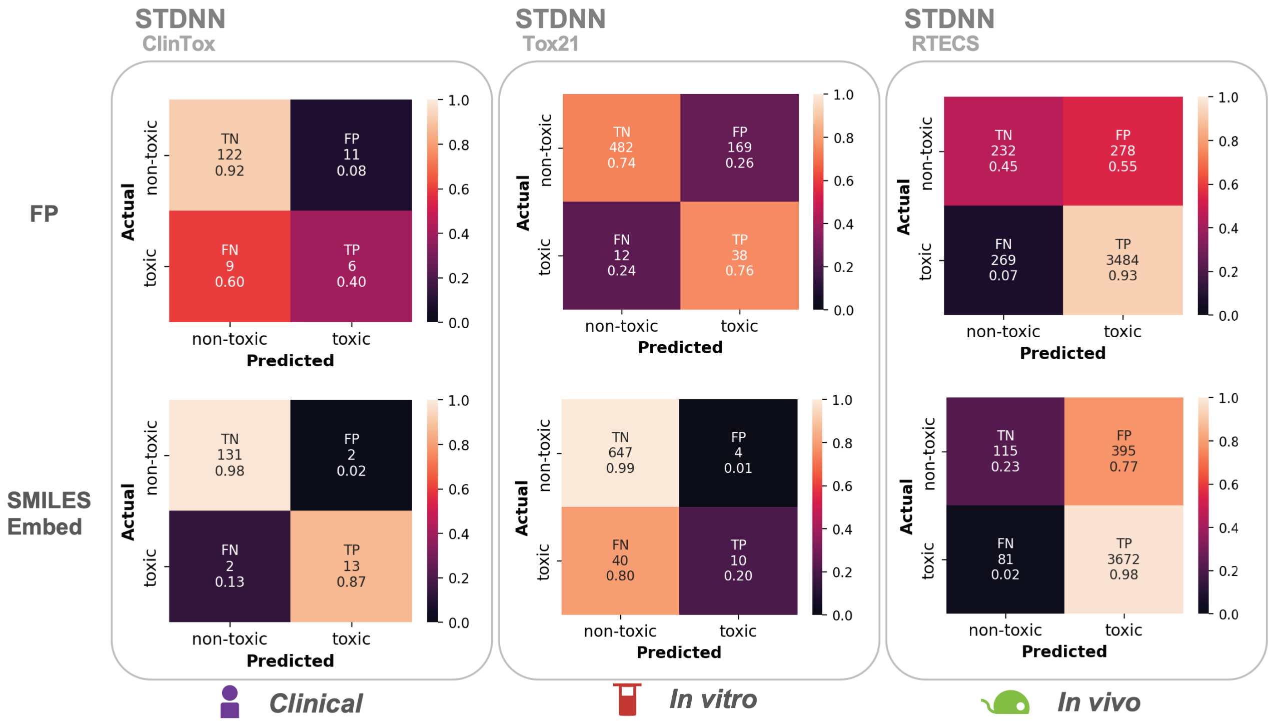

Figure S4. Confusion matrices for single-task DNN with Morgan fingerprints vs. SMILES embeddings as input, for ClinTox, Tox21 and RTECS tasks. Each quadrant of the confusion matrix provides: (1) total number of chemicals, (2) normalized fraction of, true negative (TN), false positive (FP), false negative (FN) and true positive (TP) chemicals for predictions on the test dataset for separate single-task DNNs on ClinTox, Tox21 and RTECS. Given only confusion matrix results, single-task DNN performance is better for ClinTox with SMILES embedding, for Tox21 with Morgan fingerprints and for RTECS with SMILES embeddings. At seed of 122.

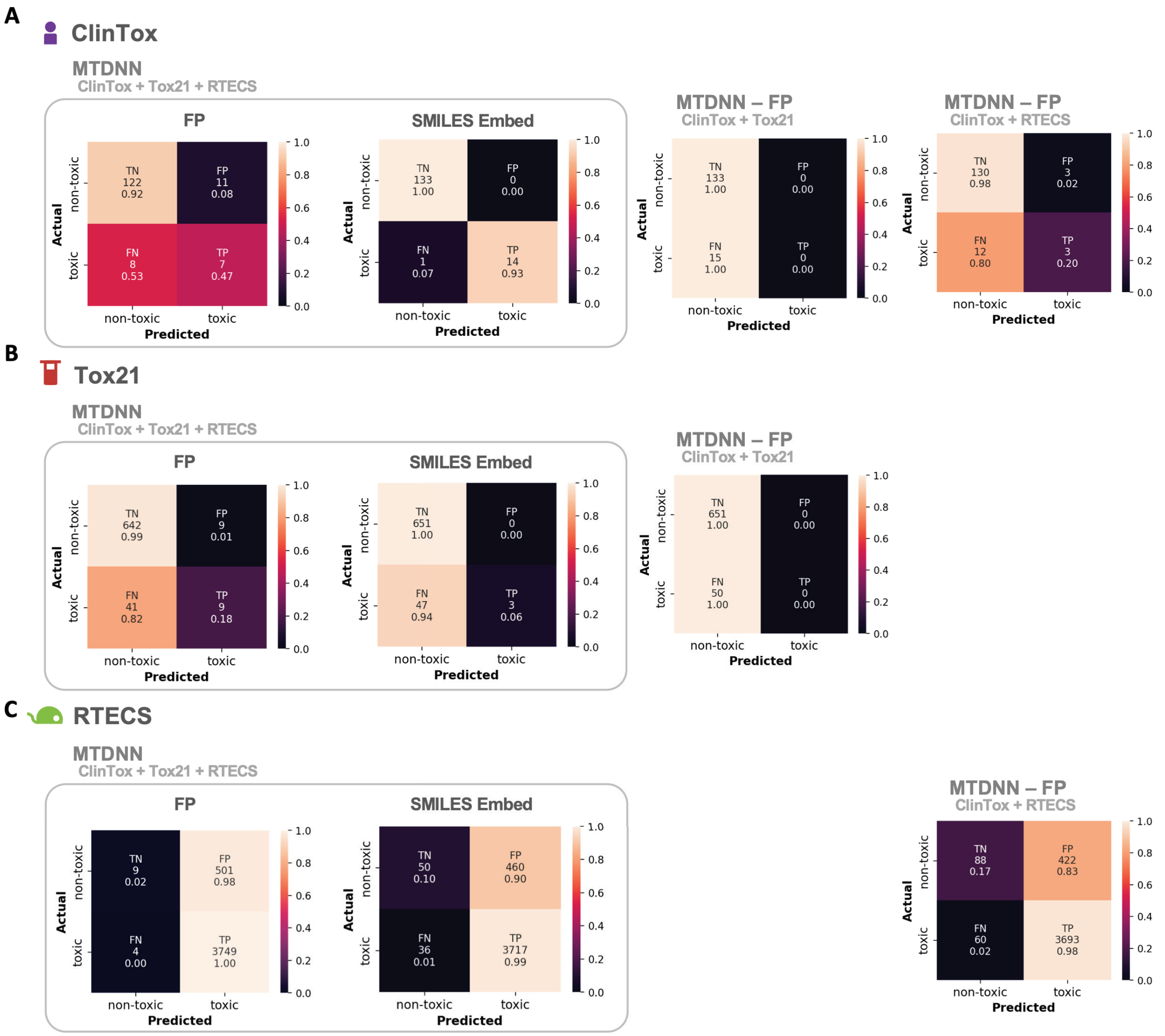

Figure S5. Confusion matrices for multi-task DNN with Morgan fingerprints vs. SMILES embeddings as input, for ClinTox, Tox21 and RTECS tasks. Models are trained on the specified datasets under the “MTDNN" label. Each quadrant of the confusion matrix provides: (1) total number of chemicals, (2) normalized fraction of, true negative (TN), false positive (FP), false negative (FN) and true positive (TP) chemicals for predictions in the specified multi-task DNN (MTDNN) tested on (a) ClinTox, (b) Tox21 and (c) RTECS. At seed of 122.

5 Cross-Confusion Matrix Comparison

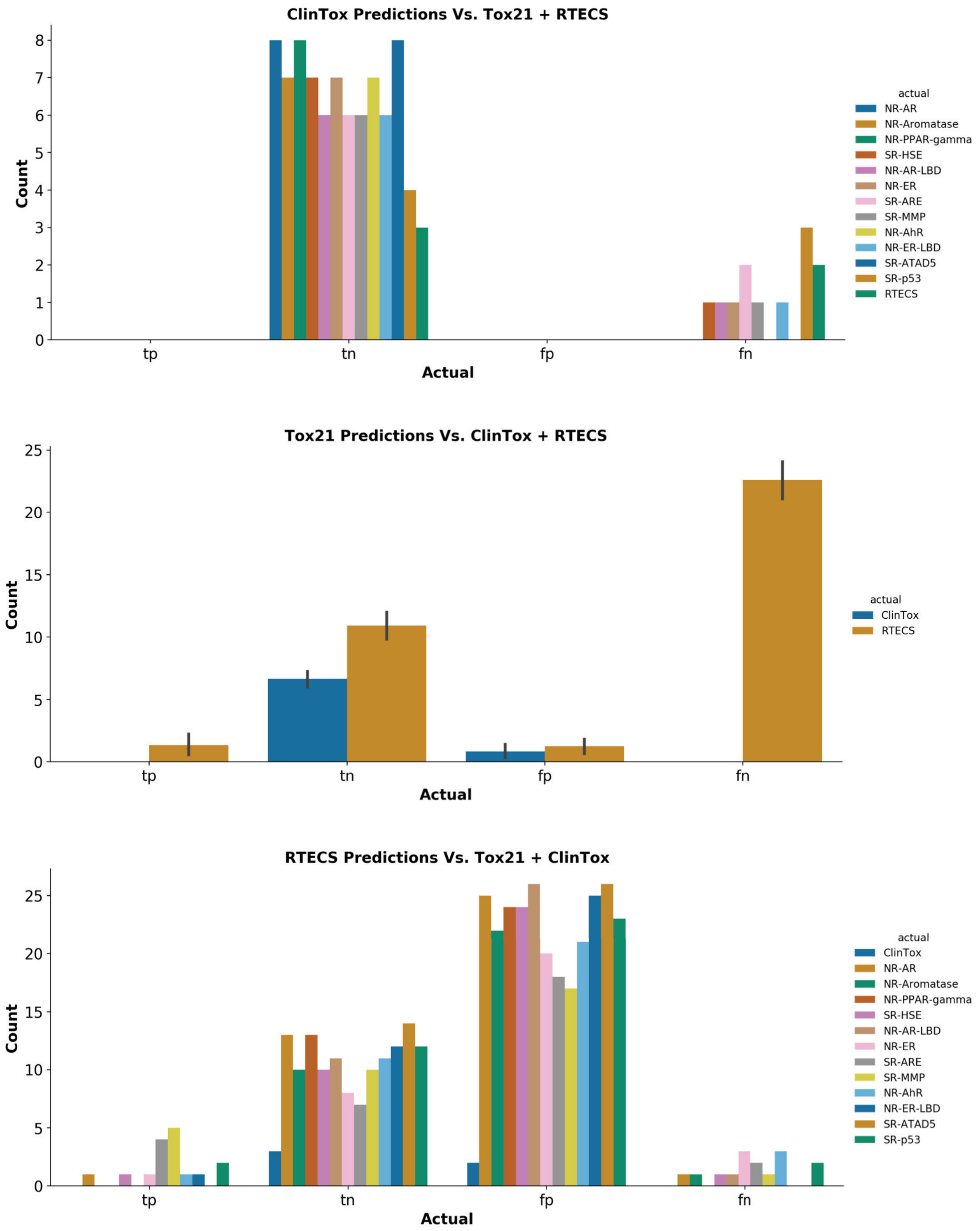

Figure S6. Distribution of true/false positives/negatives comparing ground truths *in vitro*, *in vivo* and clinically. True positive (tp), true negative (tn), false positive (fp), and false negative (fn) comparison across predictions made for Tox21 (*in vitro*), RTECS (*in vivo*) and ClinTox (clinical), using ground truths across these platforms. For instance, predictions on ClinTox are compared with ground truths given by Tox21 and RTECS datasets.

6 Full List of Top 10 Pertinent Positive and Pertinent Negative Substructures for Toxic and Nontoxic Molecules

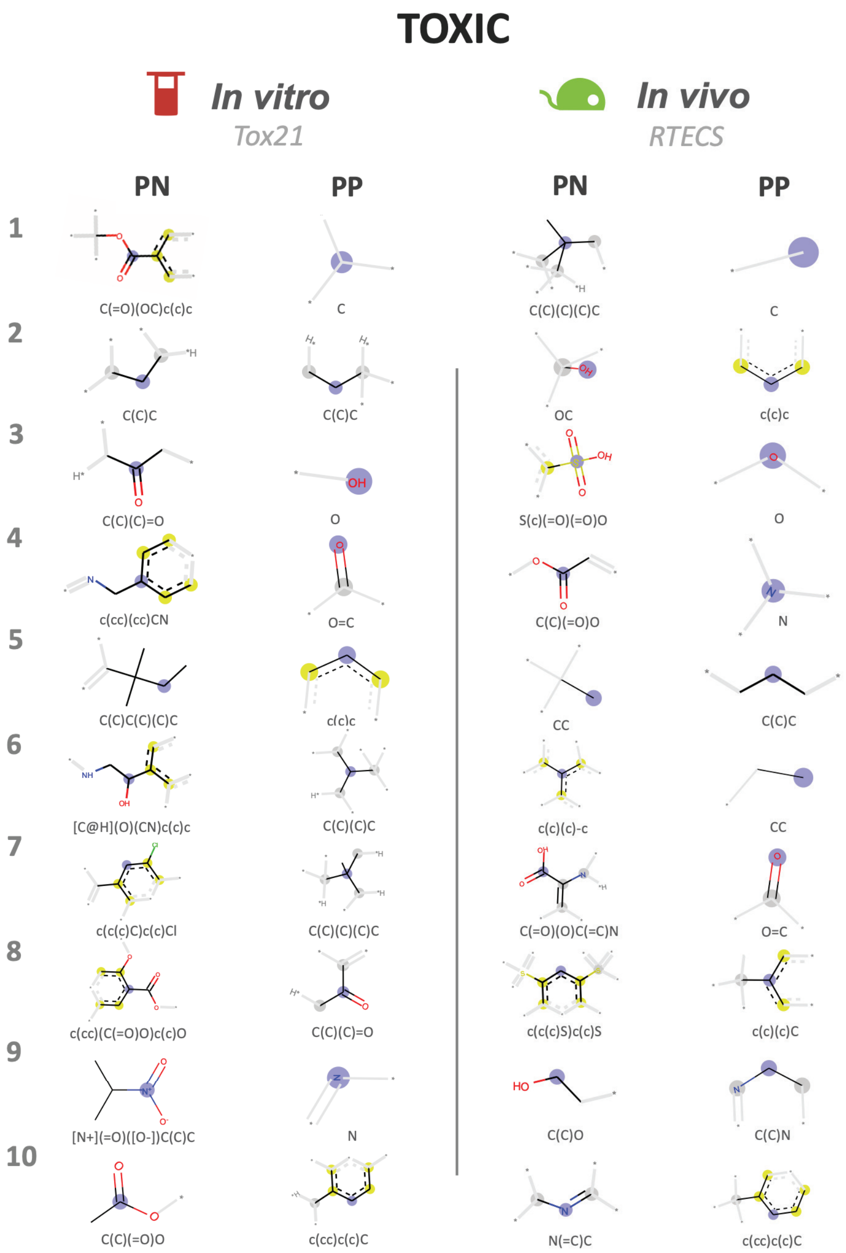

Figure S7. **Top 10 most common PP and PN substructures of correctly predicted toxic molecules for Tox21, and RTECS endpoints.** ClinTox only had 1-2 examples of toxic molecules in the test set, and was thus excluded.

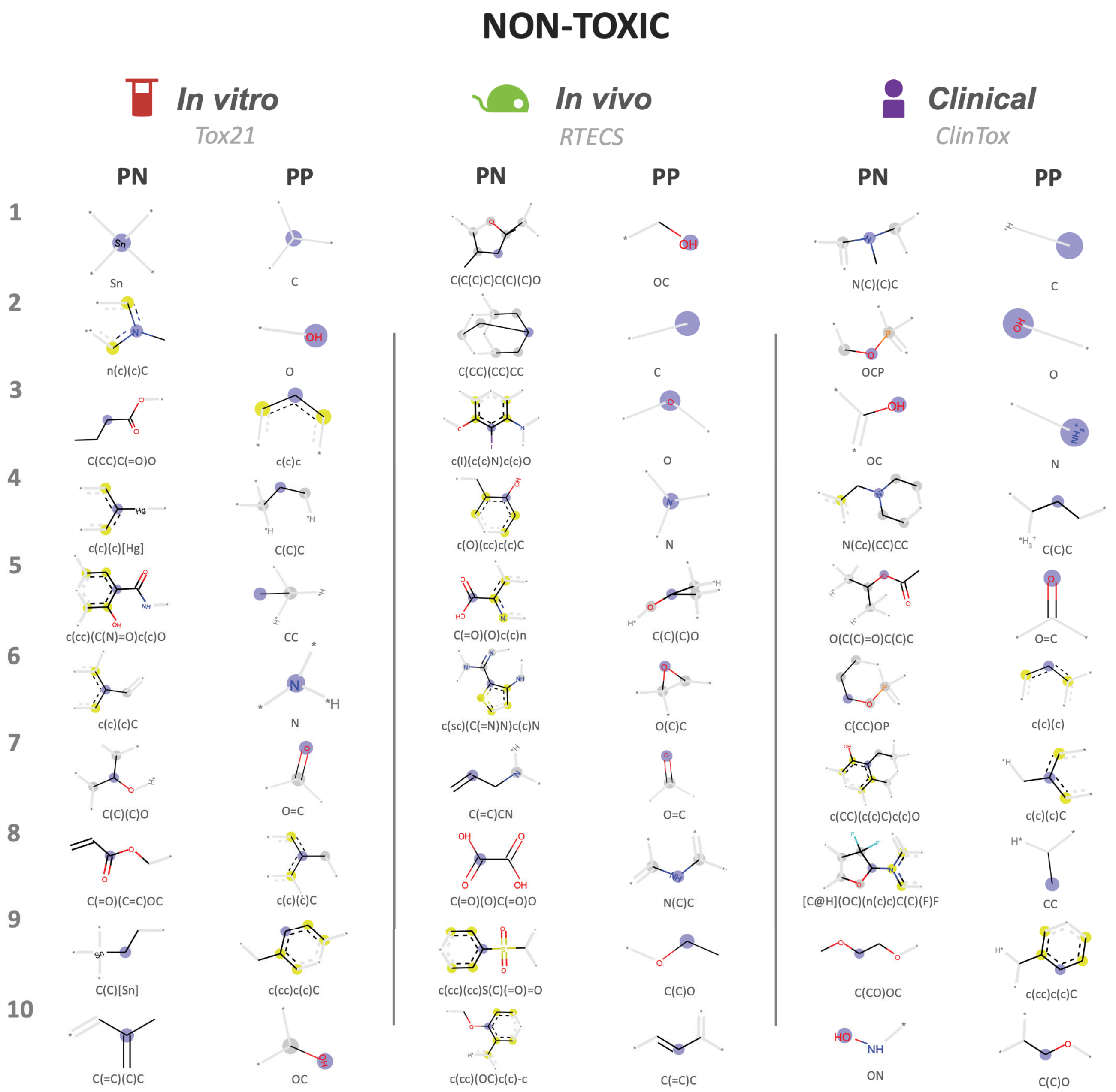

Figure S8. **Top 10 most common PP and PN substructures of correctly predicted nontoxic molecules for Tox21, ClinTox and RTECS endpoints.**

7 Match of Pertinent Features to Genetic Algorithm Features

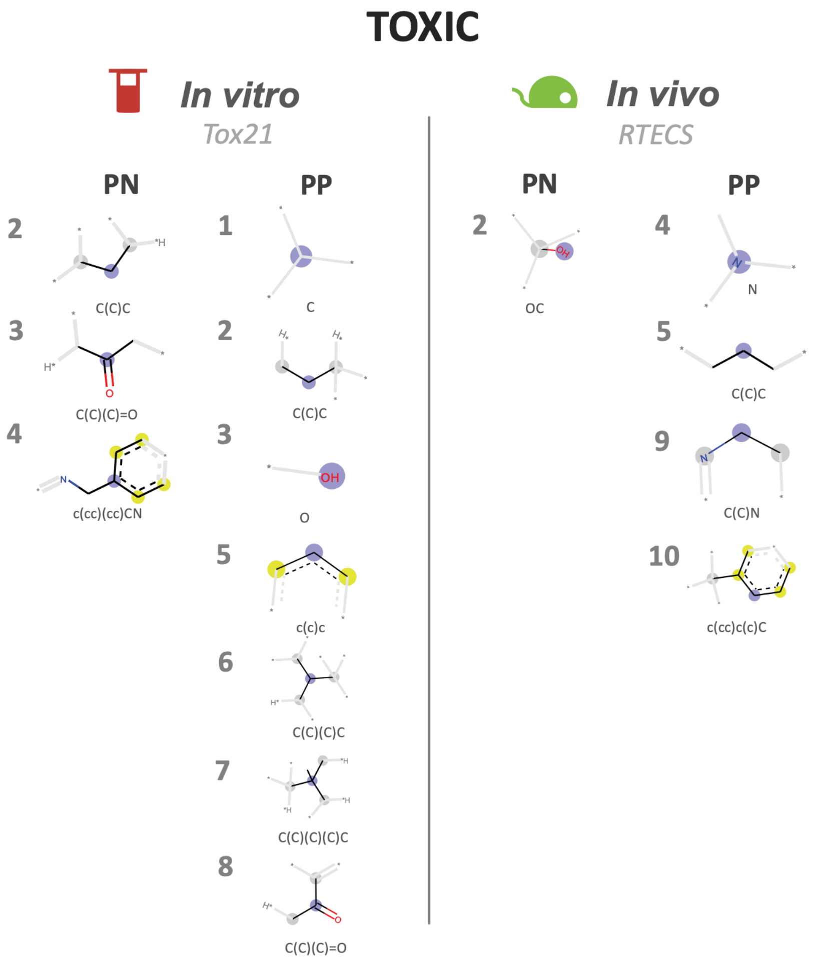

Figure S9. Features selected by the genetic algorithm that matched to the top 10 most frequent pertinent features selected by the CEM for toxic molecules. Genetic algorithm (GA) selects features to best predict an endpoint. Provided here are the features selected by the GA that were within the top 10 most frequent pertinent features of toxic molecules identified by the CEM.

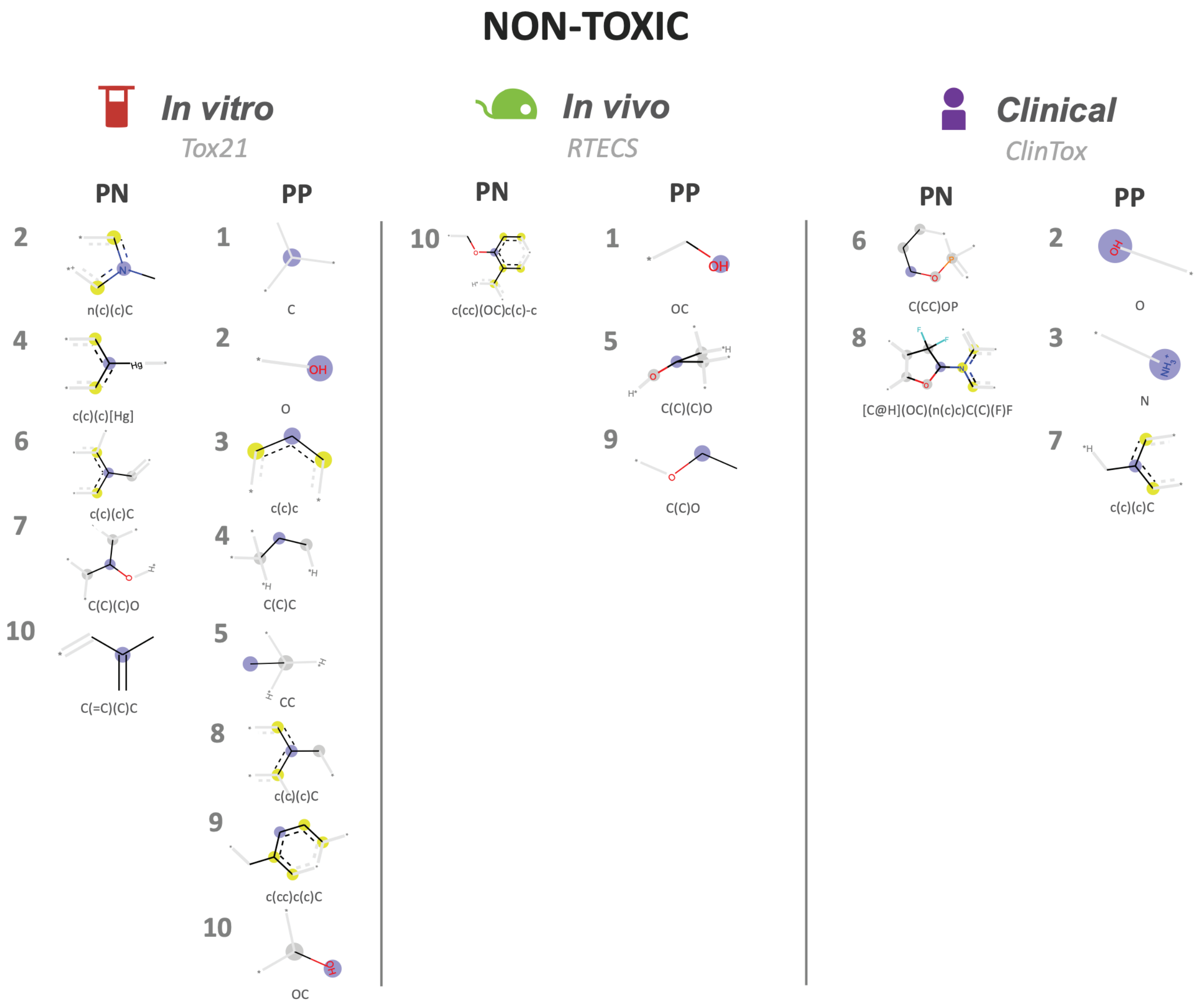

Figure S10. **Features selected by the genetic algorithm that matched to the top 10 most frequent pertinent features selected by the CEM for nontoxic molecules.** Genetic algorithm (GA) selects features to best predict an endpoint. Provided here are the features selected by the GA that were within the top 10 most frequent pertinent features of nontoxic molecules identified by the CEM.

8 Full list of Matched Toxicophores

	PP : Toxic						PN : Non-toxic					
	Mutagenic				Reactive	#	Mutagenic				Reactive	#
	experimental	#	*computational*	#			*experimental*	#	*computational*	#		
Clinical *ClinTox*							Unsubstituted heteroatom-heteroatom	42	Unsubstituted heteroatom-heteroatom	42	Michael acceptors	25
							Aromatic Amines	26	Aromatic Amine - C	29	Thioesters	4
									Aromatic Amines	26		
In Vitro *Tox21*	Aromatic Amines	20	Aromatic Amines	20	Michael acceptors	12	Aliphatic Halide	2393	Aromatic Amines (ring size =/= 3)	1544	Alkyl Halides, P/S Halides, Mustards	2003
	Unsubstituted heteroatom-heteroatom	3	Aromatic Nitro	13	Alkyl Halides, P/S Halides, Mustards	2	Aromatic Amines	1550	Unsubstituted heteroatom-heteroatom	1075	Michael Acceptors	1355
	Aliphatic Halide	2	Aromatic Amine - C	13	Thioesters	1	Unsubstituted heteroatom-heteroatom	1075	Aromatic Amines - C w/ 2 attachments	1069	Heteroatom-Heteroatom Single Bonds	321
					Reactive cyanides	1	Three-membered heterocycle	175	Aromatic Amines - C	428	Disulfides	180
							Azo-type	108	Carbonyl/Ether - Cl/Br	345	Thioesters	171
											Epoxides, Thioepoxides, Aziridines	171
In Vivo *RTECS*	Aromatic Amines	828	Aromatic Amines (ring size =/= 3)	825	Michael Acceptors	172	Aromatic Amines	68	Aromatic Amines	68	1,2-Dicarbonyls	24
	Unsubstituted heteroatom-heteroatom	357	Aromatic Amine - C	385	Alkyl Halides, P/S Halides, Mustards	158	Aliphatic Halide	19	Unsubstituted heteroatom-heteroatom	14	Alkyl Halides, P/S Halides, Mustards	18
	Aliphatic Halide	334	Unsubstituted heteroatom-heteroatom	357	Heteroatom-Heteroatom Single Bonds	145	Unsubstituted heteroatom-heteroatom	14	Aromatic Nitro	12	Michael Acceptors	9
											Heteroatom-Heteroatom Single Bonds	9

Figure S11. Matched Toxicophores. Top three (ClinTox, RTECS) or top five (Tox21) matched known toxicophores to toxicophores collected from the CEM as PP of toxic molecules and PN of nontoxic molecules. For Tox21, the top five most frequent matches were examined due to the large number of matches. Three types of known toxicophores were matched: experimental [33] and computational [34] mutagenic toxicophores , and reactive substructures [35] commonly used to filter molecules.

9 Count of Matched Toxicophores Vs. Non-Toxicophores

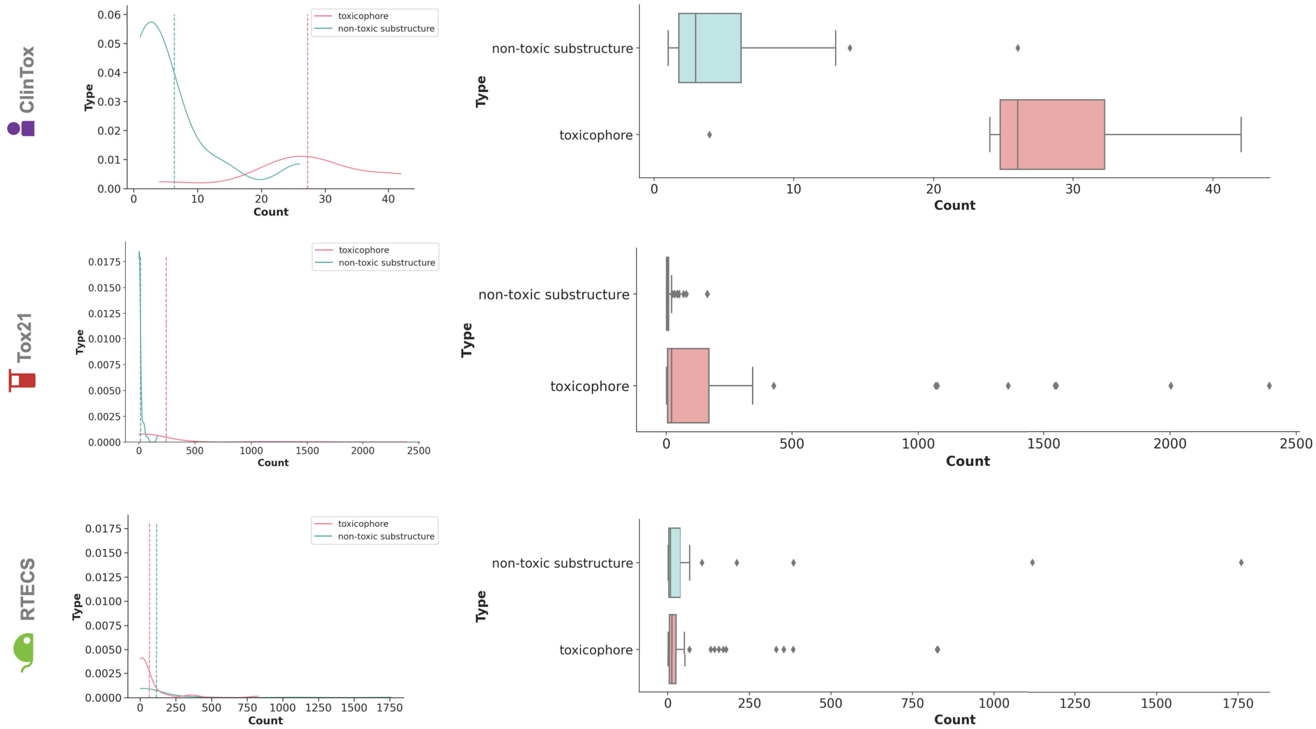

Figure S12. Count of toxicophores vs. nontoxic substructures obtained from the CEM that were matched to known toxicophores All PP and PN substructures from correct predictions were matched to known toxicophores. Out of the PP and PN substructures matched to known toxicophores, the count of substructures correlating to toxic predictions (toxicophores) was compared to the count of substructures correlating to nontoxic predictions (nontoxic substructures). Kde-plot with mean lines, and box-plot of the counts is displayed.

10 Applicability Domain

Endpoint	Maximum Variance
In vitro *Tox21*	
NR-AR	0.25
NR-Aromatase	0.27
NR-PPAR-gamma	0.22
SR-HSE	0.25
NR-AR-LBD	0.20
NR-ER	0.19
SR-ARE	0.29
SR-MMP	0.30
NR-AhR	0.27
NR-ER-LBD	0.19
SR-ATAD5	0.16
SR-p53	0.18
In vivo *RTECS*	0.28
Clinical *ClinTox*	0.21

Figure S13. Maximum variance for each endpoint defining Applicability Domain of the MTDNN-FP trained on all three platforms. Maximum variance of the predictive probabilities on the training chemicals over five randomly initialized MTDNN-FP model trained on all three platforms. Seed values of 122, 123, 124, 125, and 126 were used for each respective model. Applicability domain is defined by having a variance on a new chemical less than the provided maximum variance.

11 Model Architecture

A

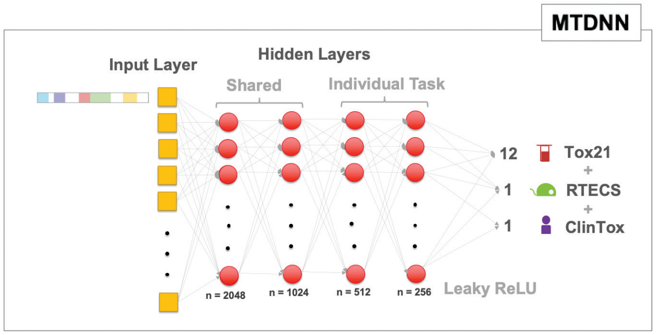

B

Multi-task DNN	
Trained on + Predicts	12 Tox21 + 1 RTECS + 1 ClinTox
Platform	PyTorch
Layers	2 shared, 2 task specific (2048, 1024, 512, 256 nodes)
Missing Labels	-1
Loss	Binary Cross Entropy Loss
Optimizer	Adam : 0.001 lr
Normalization	Batch normalization
Minibatch size	512
# of Epochs	Determined by minimum valid loss (1 – 4)
Seed	122, 123, 124

Figure S14. Model architecture. (a) Multi-task Deep Neural Network (MTDNN) base architecture. Input layer of chemical fingerprints is passed through two shared layers, and two further layers for each task. Each layer undergoes batch normalization and is activated by Leaky ReLU. The model combines prediction on Tox21 (*in vitro*), ClinTox (clinical) and RTECS (*in vivo*) endpoints. (b) Hyperparameters used.

References

1. Gadaleta, D. *et al.* SAR and QSAR modeling of a large collection of LD50 rat acute oral toxicity data. *J. Cheminformatics* **11**, 58, DOI: https://doi.org/10.1186/s13321-019-0383-2 (2019).
2. Li, X. *et al.* In silico prediction of chemical acute oral toxicity using multi-classification methods. *J. Chem. Inf. Model.* **54**, 1061–1069, DOI: https://doi.org/10.1021/ci5000467 (2014).
3. Idakwo, G. *et al.* Deep learning-based structure-activity relationship modeling for multi-category toxicity classification: A case study of 10K Tox21 chemicals with high-throughput cell-based androgen receptor bioassay data. *Front. Physiol.* **10**, DOI: https://doi.org/10.3389/fphys.2019.01044 (2019).
4. Chen, L. *et al.* Predicting chemical toxicity effects based on chemical-chemical interactions. *PloS One* **8**, e56517, DOI: https://doi.org/10.1371/journal.pone.0056517 (2013).
5. Huang, T., Jiang, Z., Xu, R. & Dong, C. Identification of chemical toxicity using ontology information of chemicals. *Comput. Math. Methods Medicine* **2015**, 246374, DOI: https://doi.org/10.1155/2015/246374 (2015).
6. Raies, A. B. & Bajic, V. B. In silico toxicology: comprehensive benchmarking of multi-label classification methods applied to chemical toxicity data. *WIREs Comput. Mol. Sci.* **8**, e1352, DOI: https://doi.org/10.1002/wcms.1352 (2018).
7. Wu, Z. *et al.* MoleculeNet: a benchmark for molecular machine learning. *Chem. Sci.* **9**, 513–530, DOI: https://doi.org/10.1039/c7sc02664a (2017).
8. Sosnin, S., Karlov, D., Tetko, I. V. & Fedorov, M. V. Comparative study of multitask toxicity modeling on a broad chemical space. *J. Chem. Inf. Model.* **59**, 1062–1072, DOI: https://doi.org/10.1021/acs.jcim.8b00685 (2019).
9. Xu, Y., Pei, J. & Lai, L. Deep learning based regression and multiclass models for acute oral toxicity prediction with automatic chemical feature extraction. *J. Chem. Inf. Model.* **57**, 2672–2685, DOI: https://doi.org/10.1021/acs.jcim.7b00244 (2017).
10. Liu, J. *et al.* Predicting hepatotoxicity using toxcast in vitro bioactivity and chemical structure. *Chem. Res. Toxicol.* **28**, 738–751, DOI: https://doi.org/10.1021/tx500501h (2015).
11. Liu, J., Patlewicz, G., Williams, A. J., Thomas, R. S. & Shah, I. Predicting organ toxicity using in vitro bioactivity data and chemical structure. *Chem. Res. Toxicol.* **30**, 2046–2059, DOI: https://doi.org/10.1021/acs.chemrestox.7b00084 (2017).
12. Abdelaziz, A., Spahn-Langguth, H., Schramm, K.-W. & Tetko, I. V. Consensus modeling for HTS assays using in silico descriptors calculates the best balanced accuracy in Tox21 challenge. *Front. Environ. Sci.* **4**, DOI: https://doi.org/10.3389/fenvs.2016.00002 (2016).

13. Mayr, A., Klambauer, G., Unterthiner, T. & Hochreiter, S. DeepTox: Toxicity prediction using deep learning. *Front. Environ. Sci.* **3**, DOI: https://doi.org/10.3389/fenvs.2015.00080 (2016).

14. Wu, Z. *et al.* MoleculeNet: a benchmark for molecular machine learning. *Chem. Sci.* **9**, 513–530, DOI: https://doi.org/10.1039/c7sc02664a (2017).

15. Pérez-Parras Toledano, J., García-Pedrajas, N. & Cerruela-García, G. Multilabel and missing label methods for binary quantitative structure–activity relationship models: An application for the prediction of adverse drug reactions. *J. Chem. Inf. Model.* **59**, 4120–4130, DOI: https://doi.org/10.1021/acs.jcim.9b00611 (2019).

16. Muñoz, E., Nováček, V. & Vandenbussche, P.-Y. Facilitating prediction of adverse drug reactions by using knowledge graphs and multi-label learning models. *Briefings Bioinforma.* **20**, 190–202, DOI: https://doi.org/10.1093/bib/bbx099 (2017).

17. Wadhwa, S., Gupta, A., Dokania, S., Kanji, R. & Bagler, G. A hierarchical anatomical classification schema for prediction of phenotypic side effects. *PLoS One* **13**, e0193959, DOI: https://doi.org/10.1371/journal.pone.0193959 (2018).

18. Jiménez-Luna, J., Grisoni, F. & Schneider, G. Drug discovery with explainable artificial intelligence. *Nat. Mach. Intell.* **2**, 573–584, DOI: https://doi.org/10.1038/s42256-020-00236-4 (2020).

19. Polishchuk, P. Interpretation of quantitative structure–activity relationship models: Past, present, and future. *J. Chem. Inf. Model.* **57**, 2618–2639, DOI: https://doi.org/10.1021/acs.jcim.7b00274 (2017).

20. Sharma, A. K., Srivastava, G. N., Roy, A. & Sharma, V. K. ToxiM: A toxicity prediction tool for small molecules developed using machine learning and chemoinformatics approaches. *Front. Pharmacol.* **8**, DOI: https://doi.org/10.3389/fphar.2017.00880 (2017).

21. Rasmussen, M. H., Christensen, D. S. & Jensen, J. H. Do machines dream of atoms? a quantitative molecular benchmark for explainable AI heatmaps. *ChemRxiv preprint* DOI: 10.26434/chemrxiv-2022-gnq3w (2022).

22. Polishchuk, P. G., Kuz'min, V. E., Artemenko, A. G. & Muratov, E. N. Universal approach for structural interpretation of qsar/qspr models. *Mol. Informatics* **32**, 843–853, DOI: https://doi.org/10.1002/minf.201300029 (2013).

23. Pope, P. E., Kolouri, S., Rostami, M., Martin, C. E. & Hoffmann, H. Explainability methods for graph convolutional neural networks. In *2019 IEEE/CVF Conference on Computer Vision and Pattern Recognition (CVPR)*, 10764–10773, DOI: https://doi.org/10.1109/CVPR.2019.01103 (2019).

24. Akita, H. *et al.* BayesGrad: Explaining predictions of graph convolutional networks. In *International Conference on Neural Information Processing*, 81–92 (Springer, 2018).

25. Jiménez-Luna, J., Skalic, M., Weskamp, N. & Schneider, G. Coloring molecules with explainable artificial intelligence for preclinical relevance assessment. *J. Chem. Inf. Model.* **61**, 1083–1094, DOI: https://doi.org/10.1021/acs.jcim.0c01344 (2021).

26. Ramsundar, B., Eastman, P., Walters, P. & Pande, V. *Deep Learning for the Life Sciences: applying deep learning to genomics, microscopy, drug discovery, and more* (O'Reilly Media, 2019).

27. Ribeiro, M. T., Singh, S. & Guestrin, C. "why should i trust you?": Explaining the predictions of any classifier. In *Proceedings of the 22nd ACM SIGKDD International Conference on Knowledge Discovery and Data Mining*, KDD '16, 1135–1144, DOI: https://doi.org/10.1145/2939672.2939778 (Association for Computing Machinery, New York, NY, USA, 2016).

28. Lundberg, S. M. & Lee, S.-I. A unified approach to interpreting model predictions. In *Proceedings of the 31st International Conference on Neural Information Processing Systems*, NIPS'17, 4768–4777 (Curran Associates Inc., Red Hook, NY, USA, 2017).

29. Rodríguez-Pérez, R. & Bajorath, J. Interpretation of compound activity predictions from complex machine learning models using local approximations and shapley values. *J. Medicinal Chem.* **63**, 8761–8777, DOI: https://doi.org/10.1021/acs.jmedchem.9b01101 (2020).

30. Jiang, D. *et al.* Could graph neural networks learn better molecular representation for drug discovery? a comparison study of descriptor-based and graph-based models. *J. Cheminformatics* **13**, 12, DOI: https://doi.org/10.1186/s13321-020-00479-8 (2021).

31. Ying, R., Bourgeois, D., You, J., Zitnik, M. & Leskovec, J. GNNExplainer: Generating Explanations for Graph Neural Networks. *arXiv e-prints* arXiv:1903.03894, DOI: https://doi.org/10.48550/arXiv.1903.03894 (2019).

32. Preuer, K., Klambauer, G., Rippmann, F., Hochreiter, S. & Unterthiner, T. *Interpretable Deep Learning in Drug Discovery.*, 331–345 (Springer International Publishing, Cham, 2019).

33. Kazius, J., McGuire, R. & Bursi, R. Derivation and validation of toxicophores for mutagenicity prediction. *J. Medicinal Chem.* **48**, 312–320, DOI: https://doi.org/10.1021/jm040835a (2005).

34. Yang, H. *et al.* Evaluation of different methods for identification of structural alerts using chemical ames mutagenicity data set as a benchmark. *Chem. Res. Toxicol.* **30**, 1355–1364, DOI: https://doi.org/10.1021/acs.chemrestox.7b00083 (2017).

35. Hevener, K. E. Computational toxicology methods in chemical library design and high-throughput screening hit validation. *Methods Mol Biol* **1800**, 275–285, DOI: https://doi.org/10.1007/978-1-4939-7899-1_13 (2018).
